# Unlocking the potential of remdesivir: innovative approaches to drug delivery

**DOI:** 10.1007/s13346-025-01843-7

**Published:** 2025-04-17

**Authors:** Maie S. Taha, Alaa Akram, Ghada A. Abdelbary

**Affiliations:** Department of Pharmaceutics and Industrial Pharmacy, Faculty of Pharmacy, Cairo University, Cairo, 11562 Egypt

**Keywords:** Remdesivir, Complex generics, Remdesivir combinations, Remdesivir challenges, Advanced drug delivery systems, Viral infections

## Abstract

Given the recurrent waves of COVID-19 and the emergence of new viral infections, optimizing the potential of remdesivir as an antiviral agent is critical. While several reviews have explored the efficacy of remdesivir, few have comprehensively addressed its challenges, such as the necessity for intravenous infusion, suboptimal lung accumulation, and safety concerns related to its formulation. This review critically examines these challenges while proposing innovative solutions and effective combinations with other antiviral agents and repurposed drugs. By highlighting the role of complex generics, we aim to enhance therapeutic efficacy in ways not previously discussed in existing literature. Furthermore, we address the development of novel drug delivery systems which specifically aim to improve remdesivir's pharmacological profile. By analyzing recent findings, we assess both the successes and limitations of current approaches, providing insights into ongoing challenges and strategies for further optimization. This review uniquely focuses on targeted drug delivery systems and innovative formulations, thereby maximizing remdesivir's therapeutic benefits and broadening its application in combating emerging viral threats. In doing so, we fill a critical gap in literature, offering a comprehensive overview that informs future research and clinical strategies.

**Graphical Abstract:**

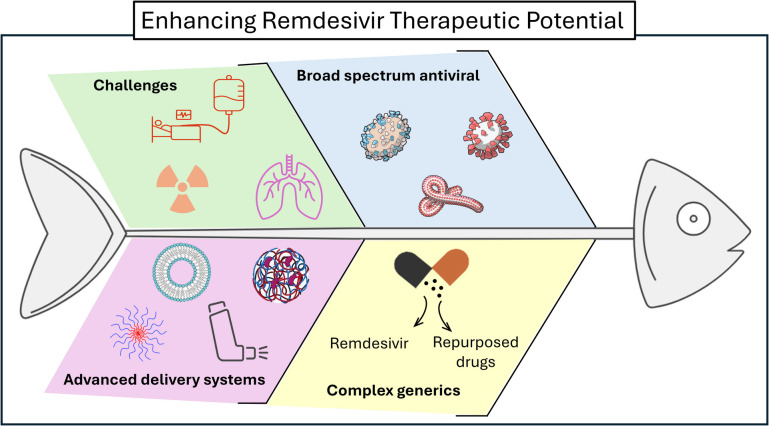

## Remdesivir, a broad spectrum antiviral prodrug

The demand for antiviral medications has surged worldwide due to the emergence of pandemic and epidemic viral infections. While vaccines are crucial for preventing the spread of viral diseases, the development of effective vaccines against mutated viruses requires extensive research and time. In the meantime, it is essential to focus on the development of antiviral drugs to address this urgent challenge [1, 2].

**Fig. 1:**
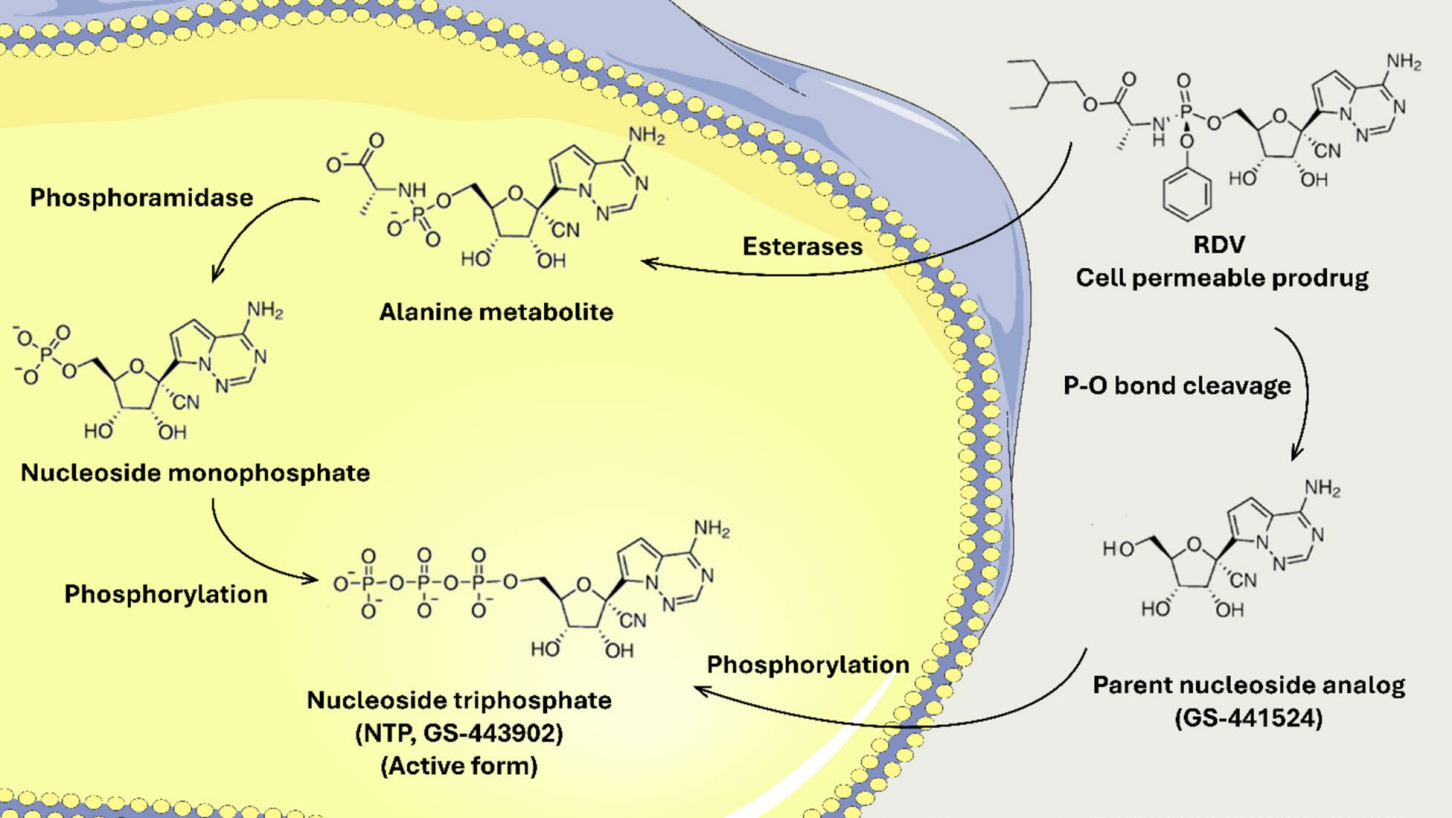
Illustrative diagram representing RDV intracellular activation

Remdesivir (RDV), developed by Gilead, is a broad-spectrum antiviral nucleotide analog that has demonstrated in vitro and in vivo antiviral activity against various RNA viruses [3–5]. RDV has been designed as a phosphoramidate prodrug to facilitate permeability into the cells (Fig. 1). The metabolism of RDV, involving a series of enzymatic conversions leading to the pharmacologically active form, is crucial for its antiviral activity against RNA viruses. Intracellularly, RDV is first converted to an alanine metabolite by esterases, then undergoes phosphoramidation to form a nucleoside monophosphate molecule. Two main metabolic pathways, phosphorylation and dephosphorylation, further modify the molecule. The phosphorylated form, known as NTP (nucleoside triphosphate), is the pharmacologically active form which competes with the natural substrate ATP in the active site of RNA-dependent RNA Polymerase. By incorporating itself into nascent RNA, RDV inhibits viral replication [6–8]. Alternatively, RDV is metabolized into its parent nucleoside analog (GS-441524) that permeates the cells, undergoing phosphorylation into the NTP active form [9]. Given the fewer metabolic steps required for GS-441524 conversion to NTP, it has an enhanced therapeutic potential.

RDV was initially developed to treat the Ebola virus during the 2018 outbreak in the eastern Democratic Republic of the Congo [2, 10]. RDV has also been studied for its effectiveness against other filoviruses, flaviviruses, and paramyxoviruses (Table 1). Unlike monoclonal antibodies, RDV is active against a wider range of viruses, thus is considered a valuable safer option with less side effects [11].

**Table 1:** Summary of RDV efficacy against various viruses

Virus	Study details	Key findings	References
Ebola virus (EBOV)	Once daily intravenous administration of 10 mg RDV/Kg for 12 days, in a rhesus monkey model of Ebola virus disease, starting 3 days post inoculation	- The pharmacologically active metabolite of RDV distributed all over the body including the brain within 4 h of administration - Suppression of virus replication as detected in plasma - Survival of 100% of infected animals - Amelioration of clinical pathological signs of the disease	[18]
Novel filoviruses (Bombali virus (BOMV), Reston virus (RESTV), and Lloviu virus (LLOV))	Reverse genetics-based minigenome systems were used to assess polymerase activity in response to RDV	- Effective inhibition in polymerase activity of BOMV, RESTV, and LLOV, with slightly lower IC_50_ values compared to EBOV	[19]
Flaviviruses (Dengue and Zika viruses, Tick-borne and Japanese encephalitis, West Nile fever, and Yellow fever)	In-vitro alkaline phosphatase-coupled polymerase assay, using a synthesized RDV triphosphate form (NTP)	- All flaviviral polymerases tested were inhibited by RDV, with IC_50_ ranging from 0.2 to 2.2 μM	[20]
Paramyxoviridae viruses (Nipah virus)	Once daily intravenous administration of 10 mg RDV/Kg for 12 days, in African green monkeys, 24 h post inoculation of a lethal dose of Nipah virus, Bangladesh genotype	- Only mild respiratory signs observed in RDV-treated animals and were resolved later - All RDV-treated animals survived 92 days post infection (the experimental period) compared to control animals which showed respiratory distress and were euthanized on days 7 and 8 post infection	[5]
MERS-CoV	Prophylactic (5 mg RDV/Kg, 24 h prior to inoculation) and therapeutic (5 mg RDV/Kg, 12 h post inoculation) RDV treatment in a rhesus macaque model of MERS-CoV infection. Treatments were administered daily until 6 days post inoculation	- Both prophylactic and therapeutic RDV treatment significantly reduced viral load in the lungs and other respiratory tissues and reduced the respiratory clinical disease score - Prophylactic RDV prevented lung lesions formation - Therapeutic RDV significantly reduced the number and severity of lung lesions compared to control group	[21]
SARS-CoV-2	Clinical studies in hospitalized patients (double-blinded randomized cohort study, Case study)	- Decreased mortality rate even with low oxygen saturation levels - More than 50% of patients showed clinical improvement with an increased recovery rate and increased hospital discharge rate - Well-tolerated in pregnant women and pediatrics with demonstrated clinical benefits, even with cancer co-morbidity	[22–29]

RDV inhibits all genotypes of the Middle East Respiratory Syndrome coronavirus (MERS-CoV) and the Severe Acute Respiratory Syndrome coronavirus (SARS-CoV-2) responsible for the COVID-19 pandemic [12]. RDV, or Veklury®, was the first FDA-approved drug for COVID-19 treatment [13]. A study comparing RDV with lopinavir, ritonavir, and interferon beta (LPV/RTV-IFNb) combination therapy showed superiority of RDV in diminishing lung viral loads and improving pulmonary function in MERS-CoV-infected mice [10]. Studies, including case studies, cohorts, and randomized controlled trials, have evaluated the safety and effectiveness of RDV in patients with COVID-19. These studies included pregnant women, pediatric cases, and immunocompromised patients [14–17].

RDV has also shown potential as an anticancer drug against glioblastoma (GBM), an aggressive intracranial malignant tumor with high recurrence and mortality rates. By inducing endoplasmic reticulum (ER) stress and activating the PERK-mediated unfolded protein response, RDV showed a superior antitumor effect compared to temozolomide commonly used for GBM treatment [30]. This effect was additionally shown in a GBM xenograft mouse model, at an 80 mg/Kg dose, which is much lower than the dose used in COVID-19 treatment. RDV metabolites can cross the blood brain barrier (BBB) [31, 32], making its use favorable for GBM therapy. RDV has also been shown to induce apoptosis in other cancer cells such as PC3 prostate cancer, HepG2 hepatocellular carcinoma, and A2058 malignant melanoma cells [33].

## Remdesivir combinations for enhanced efficacy and clinical outcomes

COVID-19 is an immune-mediated inflammatory disease resulting from SARS-CoV-2 infection. Thus, combination therapies that simultaneously alleviate inflammation and reduce viral loads are advantageous for managing the cytokine storm syndrome associated with viral clearance that can lead to organ failure [34]. Dexamethasone, a glucocorticoid recognized as a standard of care for hospitalized COVID-19 patients, has demonstrated effectiveness when combined with RDV, leading to reduced death rate or transfer to intensive care [35, 36]. Similarly, in a hamster model of SARS-CoV-2 infection, an RDV/methylprednisolone combination resulted in suppressed inflammation, antibody response, and viral loads [37]. Immunomodulators such as baricitinib and tocilizumab have also been shown to enhance recovery times and improve respiratory function when combined with RDV therapy [38, 39]. Notably, a combination of cyclosporine and RDV led to a significant reduction in IL-6 production along with decreased viral load [40]. Additionally, various other RDV combinations with repurposed drugs, summarized in Table 2, have been investigated to increase its therapeutic efficacy while minimizing doses and side effects, as well as decreasing the propensity for resistance emergence. These combinations have shown significant enhancements in antiviral activity and reduction in inflammatory cytokines production by targeting multiple viral and host processes [41–49].

**Table 2:** Summary of RDV combinations and their effects

Combination drug	Mechanism of action	Indication	Therapeutic benefits/Key findings	References
Dexamethasone	Anti-inflammatory glucocorticoid	COVID-19 patients	▪ Reduced death rate, transfer rate to intensive care units, and length of hospitalization ▪ Faster SARS-CoV-2 clearance	[35, 36]
Baricitinib/ Tocilizumab	JAK1/JAK2 inhibitors	COVID-19 patients	▪ Shorter recovery time Accelerated improvement in respiratory status ▪ Fewer side effects	[38, 39]
Disulfiram/ Ebselen	Zn ejecting agents	SARS-CoV-2	▪ Synergistic inhibition of SARS-CoV-2 replication in Vero E6 cells	[42]
Ivermectin	Inhibits viral protein nuclear translocation	Murine hepatitis virus	▪ Reduced virus replication and viral RNA synthesis ▪ Reduced IL-6, LIF and TNF-α production from infected RAW264.7 macrophages	[50]
Fluoxetine/ Itraconazole	Interfere with endolysosomal cholesterol release	SARS-CoV-2	▪ Synergistic reduction (> 90%) of SARS-CoV-2 viral titer in Calu-3 cells	[45]
Nirmatrelvir	Protease 3CL inhibitor	SARS-CoV-2	▪ Synergistic reduction of SARS-CoV-2 viral titer in Vero E6 cells	[51]
Cyclosporine	Calcineurin inhibitor	Human coronaviruses OC43 and SARS-CoV-2	▪ Synergistic reduction in viral replication and IL-6 production in infected MRC-5 and HCT-8 cells	[40]
Calpeptin	Cysteine protease inhibitor/extracellular vesicles inhibitor	SARS-CoV-2	▪ Synergistic reduction in viral replication and production of infectious virions in infected Vero E6 and Calu-3 cells	[52]
Ribavirin	Depletes intracellular GTP levels, inhibits viral RNA-dependent RNA polymerases, and induces lethal mutagenesis	SARS-CoV-2	▪ Synergistic antiviral inhibitory activity ▪ Increased viral mutations and haplotypes ▪ Diversity indices variation indicating viral quasi-species	[47]
Methylprednisolone	Anti-inflammatory glucocorticoid	SARS-CoV-2	In a hamster infection model ▪ Reduced inflammatory cells infiltration into nasal turbinate and lung tissues ▪ Suppression of viral titers in nasal turbinate, trachea and lung tissues ▪ Diminished anti-receptor-binding domain (anti-RBD) response	[53]
Velpatasvir/Sofosbuvir (Epclusa®) Elbasvir/Grazoprevir (Zepatier®)	-Hepatitis C virus nonstructural protein 5A inhibitors -SARS-CoV-2 exonuclease proofreader inhibitors	SARS-CoV-2	▪ Synergistic reduction in virus-induced cytopathic effect in Vero E6 cells ▪ Synergistic reduction in virus replication, reducing viral load in Calu-3 cells	[54]
Favipiravir	RNA-dependent RNA-polymerase inhibitor	SARS-CoV-2	In a hamster infection model ▪ Reduced virus load in lung tissue compared to either drug alone for both prophylactic and therapeutic regimens ▪ Prophylactic administration reduced inflammatory cells infiltration into lung tissue and avoided virus transmission via respiratory droplets	[55]
Gemcitabine	Nucleoside analogue; blocks DNA and RNA synthesis	SARS-CoV-2	Synergistic inhibition of viral replication in Calu-3 cells at gemcitabine to RDV ratio of 2:3 or 1:4	[56]

## Challenges facing remdesivir therapy

- **Intravenous (IV) infusion requirement:** When administered orally, RDV is subject to extensive first-pass metabolism, primarily involving CYP-mediated mono-oxidation of the phosphoramidate moiety, along with significant hydrolysis [57]. This metabolic profile underscores RDV's unsuitability for oral administration due to its inactivation and low bioavailability. Consequently, RDV is clinically administered as an IV infusion. For COVID-19 therapy, a loading dose of 200 mg RDV diluted in 0.9% saline or 5% dextrose is infused over 60 min on day 1, followed by IV maintenance doses of 100 mg daily for 5–9 days [8]. This limits RDV administration convenience, especially in resource-limited or outpatient settings, and adds an economic burden to the patients. Therefore, clinical application of RDV is limited to hospitalized patients with severe disease state. Moreover, intramuscular administration of RDV showed variable and slow release, probably due to its hydrophobic nature [58]. Exploring other more patient-friendly administration routes, such as inhalation, which allow for self-administration would greatly enhance accessibility and compliance to RDV therapy. Alternatively, delivering RDV orally via vehicles that bypass the portal vein would enhance its oral bioavailability and avoid the need for IV infusion. Orally-bioavailable lipid monophosphate prodrugs of RDV with potent antiviral efficacy against SARS-CoV-2 have been explored showing great promise for an effective oral therapy [59, 60].
- **Vehicle-induced toxicity:** RDV is available as a lyophilized powder or as an injectable solution, containing 3 g or 6 g of sulfobutylether-β-cyclodextrin (SBECD) per 100 mg of RDV, respectively. SBECD is used as a solubilizer to enhance RDV dissolution owing to its poor aqueous solubility. However, SBECD is known to accumulate in patients with renal impairment, and has been associated with renal toxicity, as well as liver necrosis [61]. Administration of RDV to patients with renal or hepatic impairments holds a potential toxicity risk. In fact, RDV is contraindicated in patients with eGFR < 30 mL/min or severe hepatic dysfunction except if its benefits outweigh its risks [62]. To mitigate the vehicle-induced toxicity, efforts have been made to develop alternative RDV formulations that improve RDV solubility and bioavailability without using toxic solubilizers. These formulations will be discussed in the next section.
- **Poor lung accumulation:** Despite the antiviral activity of RDV against respiratory viruses, its efficacy may be limited by poor accumulation in the lungs, the primary site for respiratory infections [8]. Pharmacokinetic data in rhesus monkeys has shown rapid drop in plasma RDV concentration following IV administration (fourfold decrease in less than 30 min) [18]. The hydrolysis of RDV to the polar nucleoside monophosphate hinders tissue distribution and cell penetration. Intracellular RDV penetration is essential to undergo phosphorylation into the active antiviral nucleoside triphosphate (NTP) which is then entrapped within cells owing to its polar nature. Despite detection of NTP in peripheral blood mononuclear cells (PBMCs), it is not known if this correlates with high intracellular lung accumulation [8]. Notably, the nucleoside analog GS-441524, has been measured in PBMCs for over 24 h following RDV administration, yet the NTP lung accumulation was low [63]. Similarly, when GS-441524 was directly administered in Rhesus monkeys, NTP accumulation in the lungs corresponded to only 4% of the administered dose [57]. To address this challenge, RDV inhalable formulations need to be developed for direct access to the main site of infection. Inhalable antiviral formulations have been shown to enhance drug accumulation in deep lung tissues, where viruses such as SARS-CoV-2 may reside [64, 65]. Accordingly, this localized delivery method can improve the therapeutic efficacy with a lower dose, minimizing potential side effects [66]. Apart from inhalable formulations, another strategy involves the use of systemically-administered nanoparticles that extend RDV circulation to enable its lung delivery. Moreover, nanoparticles’ surface can be functionalized with targeting moieties to enhance tissue as well as intracellular delivery in the lung. [8].

## Drug delivery systems of remdesivir

RDV exhibits several physicochemical properties that influence its formulation and delivery. It has a molecular weight of approximately 602.6 g/mol and exhibits poor aqueous solubility that varies depending on pH, with higher solubility in acidic conditions [67]. The log P value of RDV is approximately 2.01, suggesting moderate lipid solubility, which can impact its absorption and distribution [67]. According to the biopharmaceutics classification system (BCS), RDV is considered class II [68]. RDV has a pKa of 10.93 (strongest acid) and −2.22 (strongest base), showing 99.85% protonation at physiological pH in molecular docking simulation [32, 69]. Stability studies have shown that RDV solution for infusion is stable for only 24 h at room temperature or 48 h in the refrigerator [67]. These physicochemical characteristics underscore the need for innovative drug delivery systems to enhance RDV's therapeutic profile, addressing issues of solubility, bioavailability, and stability, thereby supporting its efficacy in treating viral infections.

**Fig. 2:**
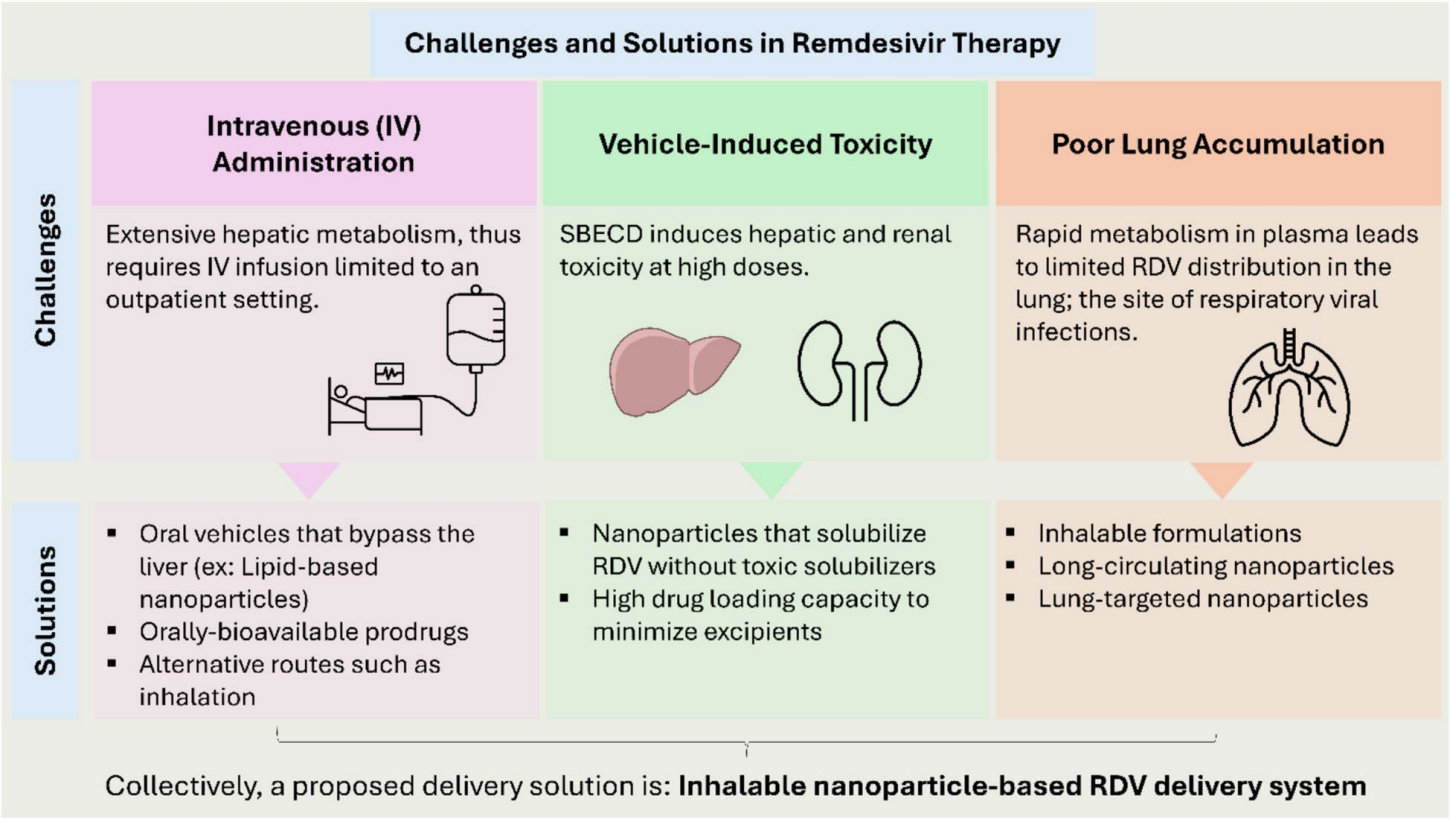
A conceptual diagram summarizing main challenges associated with RDV therapy and potential solutions or strategies to overcome these challenges

Despite its potential benefits, the World Health Organization (WHO) has cautioned against RDV use due to insufficient clinical data supporting its therapeutic efficacy [70]. This highlights the ongoing need for addressing the limitations of RDV in clinical applications to fully harness its potential as an antiviral agent against respiratory viral infections (Fig. 2).

To overcome the above mentioned hurdles and optimize RDV delivery, various drug delivery systems have been investigated. This section explores the strategies and formulations implemented to enhance bioavailability, reduce toxicity, and improve lung targeting of RDV for more effective treatment outcomes. Table 3 summarizes the developed RDV formulations and their key features.

**Table 3:** Overview of delivery systems developed for RDV: Summary of research findings

RDV delivery system	Used technique	Key findings	References
Liposomes	Thin film hydration method	▪ Sustained release of RDV from DPPC liposomes over 20 h ▪ Aerosolized RDV-loaded liposomes showed a 100-fold increase in lung levels of NTP compared to intravenous cyclodextrin solutions ▪ T_max_ was 1 h for liposomes, compared to 4 h for aerosolized RDV solutions, indicating faster bioavailability ▪ Inhalation of cyclodextrin-based RDV increased proinflammatory cytokines in the lung homogenate	[77]
	Ethanol injection method and modified hydration method	▪ DPPC failed to load RDV and showed precipitation over time ▪ DOPC led to 100% RDV encapsulation ▪ Ethanol injection method led to rapid RDV precipitation, while forming the lipid bilayer over mannitol particles prior to hydration promoted RDV encapsulation ▪ Minimal cytotoxicity of DOPC liposomes unlike DPPC liposomes against A549 lung carcinoma cells	[79]
	Thin film hydration followed by layer-by-layer coating technique	▪ Liposomes were coated with chitosan, hyaluronic acid, and a high-affinity aptamer targeting the receptor-binding domain of the spike protein of HCoV-43 ▪ Coated liposomes retained 50% of their RDV content after 120 h of release ▪ The coated liposomes did not exhibit antiviral activity against HCoV-OC43, unlike free chitosan and hyaluronic acid, which reduced viral titers by 3.25 and 2.5 log reductions, respectively	[85]
	Dehydration-rehydration technique and ethanol injection method	▪ Ethanol injection yielded smaller particles size, higher RDV encapsulation, and more sustained release ▪ Intranasal administration resulted in 100% survival of K18-hACE2 mice infected with SARS-CoV-2, reduced viral loads in both the brain and lungs, and reduced the inflammatory response induced by the virus in the lungs	[82]
Dry powder inhalation (DPI)	Thin film freezing	▪ Leucine and Captisol® DPI yielded smaller nanoaggregates compared to mannitol and lactose ▪ Higher viscosity solvent systems produced more uniformly dispersed powders ▪ Leucine-based formulations exhibited superior aerosol performance, with high FPF and low MMAD ▪ A pharmacokinetic study in Syrian hamsters demonstrated that RDV/Captisol® DPI achieved faster and greater absorption of RDV in the lungs	[125, 129]
	Spray drying	▪ Co-spraying leucine and RDV resulted in crystalline particles, with spherical morphology, and ~ 1% residual solvent ▪ The spray-dried DPI possessed inhalable particle sizes, with FPF and emitted dose exceeding 60% and 88%, respectively ▪ The DPI showed minimal toxicity to lung cells and retained the antiviral activity against SARS-CoV-2 in vitro	[131, 135]
	Nanoprecipitation with spray drying	▪ RDV nanoparticles stabilized with a PEGylated form of vitamin E succinate (TPGS) or polycaproloctone nanoparticles stabilized with Pluronic F-127 were prepared using nanoprecipitation ▪ RDV nanosuspension co-sprayed with methyl cellulose or hyaluronic acid and mannitol yielded amorphous DPI with MMAD of < 5 μm, ▪ RDV nanosuspension reconstituted from the DPI had minimal cytotoxicity, high uptake into A549 cells, and antiviral activity against SARS-CoV-2 in vitro	[138, 139]
Cocrystal-based DPI	Liquid-assisted grinding and thermal annealing followed by spray drying	▪ Salicylic acid was selected as a conformer based on the formation of strong hydrogen bonding between its carboxylic group and the triazine ring in RDV ▪ RDV/salicylic acid ratio of 10/2.3 by weight was achieved in the cocrystal ▪ The cocrystal had 14-fold faster dissolution in simulated lung fluid than unprocessed RDV	[136]
Nanocrystal-based nebulizer	Nanoprecipitation followed by lyophilization	▪ RDV nanocrystals stabilized with Pluronic F-127 (average size of 341 nm) precipitated after 30 min of aqueous reconstitution leading to inconsistent drug output upon aerosolization ▪ RDV nanocrystals with a novel triblock copolymer possessing a poly(2-oxazoline) backbone (size of 400 to 500 nm) were stable for over 25 days in solution ▪ High RDV content of 95% ▪ Aerosolization via air jet nebulizer yielded respirable particles with MMAD of around 5 µm and minimal foaming	[118]
Poly (lactic-co-glycolic acid) (PLGA) nanoparticles	Molecular docking	▪ Lisinopril was covalently grafted to PLGA, which was then employed to encapsulate RDV, for co-delivery approach ▪ Using dissipative particle dynamics simulations, optimal carrier/drug ratios of 10:1 or 5:1 were determined, resulting in spherical nanoparticles with RDV in hydrophobic core and lisinopril in hydrophilic shell	[102]
Poly (lactic-co-glycolic acid) (PLGA) long-acting injectable	Diffusion of N-methyl-2-pyrrolidone (NMP) upon injection, facilitating rapid phase separation of PLGA and soft gel depot formation	▪ Resomer 752H at concentration of 15% resulted in the highest drug loading capacity of 25% ▪ Sustained RDV release over 48 h, with a favorable burst release of 36% for an initial loading dose ▪ Ease of injectability through 25G needles with low injection force ▪ Potential for reducing dosing frequency from 5–8 intravenous doses to 2–3 subcutaneous injections	[104]
Fe(III)-doped mesoporous silica nanoparticles (mSiO2)	Reverse microemulsion method	▪ Using RDV as a template during nanoparticle formation was more effective for loading than incubation with RDV post-nanoparticles formation ▪ Enhanced degradability due to Fe(III) doping	[114]
Self-nanoemulsifying drug delivery system (SNEDDS)	Self-emulsification of bioactive oils (blackseed oil and fenugreek seed oil), surfactant (Tween 80), and cosurfactant (Imwitor 988) at ratios optimized via a pseudo ternary phase diagram	▪ The system combined RDV with baricitinib to serve as oral anticancer therapy ▪ Tween 80 and Imwitor 988 produced fine droplets stable to dilution ▪ Blackseed oil showed higher RDV solubilization efficiency and lower IC50 against MDA-MB-231 breast cancer cells and A549 lung cancer cells, compared to fenugreek seed oil	[89]
	Self-emulsification of oils (sesame oil, alpha-tocopherol, or sunflower oil), surfactant (Tween 80 or Tween 20), and cosurfactant (PEG 400 or glycerin) at ratios optimized via a pseudo ternary phase diagram	▪ The optimal formulation contained sesame oil, tween 80, and PEG 400, at a surfactant: co-surfactant ratio of 5:1 ▪ High stability to dilution without any signs of precipitation or phase separation ▪ 58% reduction in hCoV-19 viral titer compared to untreated control	[88]
Poloxamer-stabilized cyclodextrin nanoparticles	Nanoprecipitation followed by ultrasonication	▪ β-cyclodextrin formed an inclusion complex with RDV in the core, and Poloxamer 407 stabilized the nanoparticles surface against aggregation ▪ RDV solubility in pH 6.8 buffer was significantly enhanced ▪ In OVA-albumin inflammatory mouse model, the nasal spray of RDV nanoparticles significantly reduced IL-4 mRNA plasma levels compared to intravenously administered RDV ▪ Safety of RDV nanoparticles was confirmed in freshly resected goat nasal mucosa compared to a mucociliary toxic substance	[105]
Nanostructured lipid carriers (NLCs)	Hot melting technique	▪ Glyceryl monostearate, Capmul MCM, and Myrj52, were used as solid lipid, liquid lipid, and surfactant, respectively ▪ Sustained RDV release for over 48 h ▪ Ten-fold higher in vitro antiviral activity than free RDV against SARS-CoV-2 in Vero E6 cells ▪ Higher in vitro cell uptake and retention compared to free RDV ▪ A pharmacokinetic study in rats showed that NLCs increased RDV bioavailability by two folds	[92]
	Hot emulsification followed by ultrasonication	▪ Co-loading RDV and dexamethasone using Capryol 90 and Transcutol P ▪ High solubilizing power for RDV (170 mg/g) and for dexamethasone (22 mg/g) ▪ Ex vivo intratracheal administration demonstrated prolonged lung retention ▪ In vivo testing in LPS inflammatory mouse model resulted in significant reduction in proinflammatory cytokines	[94]
Chitosan-stabilized oil capsules	An internal oil core comprising Miglyol 812 N was encased in a lecithin layer followed by layer-by layer complexation with chitosan and RDV-SBECD complexes via electrostatic interactions	▪ The highest RDV content (5.6%) was achieved with low molecular weight chitosan ▪ Medium molecular weight chitosan exhibited superior control over drug release and demonstrated potent inhibition of the HCoV-OC43 replication cycle (~ 735 fold reduction in IC50) ▪ A high affinity aptamer for HCoV-O43 spike glycoproteins was attached to the nanoparticles surface via electrostatic attraction	[97]
Dendritic-linear-dendritic hyperbranched nano-scaffold	Oil-in-water (O/W) solvent evaporation method, using bis-MPA blocks linked via ester bonds to a central hydrophilic PEG chain	▪ RDV-to-dendrimer molar ratio of 1:1.5 was used to enable optimal entrapment of the drug (RDV content reached 14%) and prevent precipitation ▪ RDV encapsulation is mediated via hydrogen bonding and van der Waals interaction with the dendrimer chains ▪ PEG chains create a stealth coating around the nanoparticles, inducing steric repulsion despite a low surface charge ▪ No cytotoxicity in MRC-5 and NR8383 cells	[109]
Nanofibers	Electrospinning of SBECD-RDV solution	▪ Molecular entrapment of RDV inside SBCED turns it from crystalline to amorphous state ▪ Electrospun nano-fiber form has a superior dissolution and wettability than the lyophilized form	[140]

Nano-sized carriers offer several advantages owing to their large surface area-to-volume ratio that overcome challenges associated with conventional drug formulations. Drug encapsulation within nanoparticles increase solubility for poorly soluble drugs and confers metabolic stability shielding drugs from enzymatic degradation. This is particularly relevant for orally administered RDV, given its susceptibility to extensive first-pass metabolism. This characteristic allows for the potential enhancement of oral bioavailability, making nanoparticles a viable option for oral administration. Lipid-based nanoparticles, for instance, can leverage lymphatic transport to bypass hepatic metabolism, thus maintaining RDV stability during oral delivery. In addition, nanoparticles can be tailored to achieve sustained, controlled, or stimuli-responsive drug delivery based on the desired therapeutic profile. This feature is particularly important for RDV that requires targeted and prolonged action within specific physiological compartments for managing complex viral infections such as COVID-19. Moreover, the surface of nanoparticles can be functionalized with a variety of ligands to enable targeted delivery to specific tissues or cells. Additionally, nanoparticle-based inhalations can effectively deposit in the epithelial lining fluid, offering protection from mucociliary clearance as well as alveolar macrophages, and thus facilitate improved interactions with target cells [71].

Additionally, to achieve the potential benefits of the aforementioned RDV combinations, thoughtful drug delivery systems are needed to accommodate the different physicochemical properties of the various drug molecules. Co-loading multiple agents within a single nanoparticle system can unify their pharmacokinetics, ensuring their spatial delivery for a synergistically effect [72].

## Liposomes

Liposomes are vesicular systems composed of an aqueous core surrounded by a phospholipid bilayer. Its unique structure enables encapsulation of both hydrophilic and hydrophobic drug moieties. The fluidity of the lipid bilayers could be modified to exhibit sustained release [73], this would be beneficial for decreasing dosing schedules. Being an analog for the cell membrane makes it superiorly biocompatible compared with polymeric nanoparticles. Liposomes-based carriers have been extensively investigated for pulmonary drug delivery [74].

Pegylated dipalmitoyl phosphatidylcholine (DPPC) liposomes have been used to load RDV owing to its biocompatibility, where DPPC constitutes almost 90% of the alveolar tissue surfactants [75]. This was also thought of as a mean for enhancing uptake and retention within the lung tissue by opening the epithelial cells [76]. The liposomal system had an RDV loading content of 11.1%, an average size of 120 nm, and a sustained release over 20 h [77]. It was lyophilized with 5% trehalose and administered as an aerosolized suspension to BALB/c mice in a pharmacokinetics and biodistribution study. The liposomal system exhibited an overall 100 fold and 77 fold increase in NTP (the active RDV metabolite) levels in the lungs compared to the cyclodextrin-based RDV solution intravenously injected or aerosolized, respectively. Even 24 h post administration, RDV liposomes still exhibited 13 fold higher lung accumulation of NTP compared to intravenously injected solution. While plasma levels of RDV were not measured in this study, it is possible that the pegylated liposomal surface resulted in an extended circulation profile, contributing to increased accumulation in the lungs, a characteristic commonly associated with pegylated nanoparticles [78]. A T_max_ of 1 h post administration was observed for the RDV-loaded liposomes in contrast to 4 h with the aerosolized RDV solution, signifying the importance of nanoparticle-mediated cell uptake in addition to the localized lung administration. In addition, an increase in the proinflammatory cytokines, IL-6, TNF-α, and HMGB-1, found in the lung homogenate following inhalation of cyclodextrin-based RDV solution confirms the toxicity of this solvent system and underscores the importance of nanoparticles as alternative solubilization and delivery vehicles for RDV.

On the contrary, Vartak et al. failed to load RDV stably in DPPC liposomes observing RDV precipitation over time [79]. A combination of DPPC and dioleoyl phosphatidylcholine (DOPC) also showed instability. DOPC alone successfully loaded RDV with an almost 100% encapsulation efficiency, however the RDV loading content was not indicated. This disparate loading stability was attributed to the unsaturation in the DOPC molecule which imparted fluidity to the bilayer enabling efficient RDV loading, in contrast to the rigid bilayer of saturated DPPC [80]. It is noteworthy that the RDV liposomes were prepared using a modified hydration technique, by forming the lipid bilayer over mannitol particles prior to the hydration step. It was assumed that the mannitol osmotic pressure forced RDV encapsulation in the inner bilayers rather than the outer ones, thereby the increased encapsulation and release stability observed [81]. On the other hand, the ethanol injection method showed rapid RDV precipitation, probably due to rapid diffusion of water in the ethanolic phase and poor solubility of RDV in this mixture. Apart from their physical instability, DPPC liposomes showed significant cytotoxicity to A549 lung carcinoma cells, while DOPC showed minimal toxicity.

A dehydration-rehydration technique has been attempted for loading RDV into liposomes in a recent study, by first formulating RDV as a cyclodextrin inclusion complex before homogenizing it with blank liposomes [82]. However, compared to RDV liposomes prepared using the conventional ethanol injection method, this approach yielded larger particles sizes, lower RDV encapsulation, and a faster release profile. The optimized RDV liposomes were administered intranasally to transgenic K18-hACE2 mice infected with SARS-CoV-2, resulting in 100% survival and a significant reduction in viral loads in both the brain and lungs [82]. In contrast, the commercial formulation Veklury® demonstrated lower efficacy, with a median survival of only 8 days and limited impact on viral titers in the brain. Reducing viral loads in the brain is critical, as it helps prevent long COVID-related brain fog caused by viral invasion and persistence in the brain, which can lead to cognitive dysfunctions [83]. In this context, nose-to-brain drug delivery emerges as a highly promising approach allowing direct transport of RDV to the central nervous system (CNS), bypassing the BBB [84].

Milkova et al. aimed to improve the stability of RDV liposomes by employing alternating polyelectrolyte layers utilizing the layer-by-layer technique [85]. They coated the liposomes with chitosan, hyaluronic acid, and a high-affinity aptamer targeting the receptor-binding domain (RBD) of the spike protein of human coronavirus HCoV-43. Initially, the positively charged chitosan was intended to adhere to the negatively charged lipid bilayer. Subsequently, a layer of negatively charged hyaluronic acid was applied, followed by another layer of chitosan, facilitating the attachment of the negatively charged aptamer to the surface. The coated liposomes exhibited high stability, retaining 50% of its RDV content after 120 h of release. Despite this stability, the aptamer-functionalized liposomes did not demonstrate antiviral activity against HCoV-OC43. In contrast, free chitosan and hyaluronic acid showed antiviral effects against HCoV-OC43, reducing viral titers by 3.25 and 2.5 log reduction, respectively. The interaction between the positively charged chitosan and the negatively charged virus protective membrane disrupting it likely contributed to this antiviral activity. The failure of the chitosan-coated liposomes to show similar effect was attributed by the authors to the limited effective surface area of the chitosan coat for interaction compared to free chitosan [85]. The study lacked mechanistic exploration of the aptamer's impact on spike protein binding. Further investigation into the aptamer's conformation and interaction with the chitosan layer is warranted to better understand these findings. The sustained release of RDV might be a contributing factor to the observed lack of efficacy as well. This highlights the critical need to optimize the rate of drug release to maintain a therapeutic concentration over time.

## Self-nano-emulsifying drug delivery systems (SNEDDs)

SNEDDs are anhydrous lipid-based systems composed of oils, surfactant, and co-surfactants. Upon dilution in water, they form thermodynamically stable nanoemulsions dissolving lipophilic drugs with high capacity. SNEDDs have emerged as strategies to enhance oral delivery of drugs by increasing solubility, stability, and permeability in the gastrointestinal tract, thus enhancing their bioavailability [86]. SNEDDs have also been used for transdermal delivery where upon dilution at skin surface, supersaturated systems form acting as driving force for permeation [87].

The selection of self-emulsifying oils takes into account the solubilization power as well as the route of administration. The selection of surfactant and cosurfactant is also crucial to maintain system stability and flexibility of the interfacial layer allowing for high oil entrapment, respectively. To formulate RDV into a subcutaneous (SC) SNEDDS, sesame oil, alpha-tocopherol, and sunflower oils were tested [88]. All oils showed comparable RDV solubilization, however sesame oil was selected due to its good safety profile, well-established for SC injections. Tween 80 was selected over Tween 20, owing to the former’s lower hydrophilic-lipophilic balance (HLB) value. Similarly, PEG 400 was selected as a cosurfactant over glycerin due to significantly higher RDV solubilization power. The sesame oil: Tween 80: PEG 400 ratio was optimized via a pseudo ternary phase diagram, obtaining clear, transparent nanoemulsions showing high stability to dilution without any signs of precipitation or phase separation. The increased RDV solubility mediated by the SNEDDS was reflected in the dialysis bag study in comparison with aqueous RDV suspension, where 97% of RDV was released after 8 h compared to 17%, respectively. RDV nanoemulsion showed a 58% reduction in hCoV-19 viral titer compared to the untreated control. The authors assume an antiviral effect of the sesame oil, highlighting the added value using biologically active oils for SNEDDS preparation. Additionally, they boast the high loading capacity, small droplet size, and large surface area-to-volume ratio as mediators for increased interaction with viral membrane proteins [88]. However, a comparison with free RDV was not made, thus the actual SNEDDS advantage cannot be confirmed.

In a repurposing study, a self-nanoemulsifying drug delivery system (SNEDDS) was developed for combining RDV with baricitinib to serve as oral anticancer therapy [89]. The bioactive oils: blackseed oil and fenugreek seed oil, were used for SNEDDS development to capitalize on their antioxidant, anti-inflammatory, and potentially anticancer activity. Tween 80 and Imwitor 988 were added to the oils to produce fine droplets stable to dilution. The SNEDDs based on blackseed oil showed the highest RDV solubilization efficiency of 10.93 mg/g, with an average droplet size and surface charge of 247 nm and + 38.73 mV, respectively. The IC50 of the combination system against MDA-MB-231 breast cancer cells and A549 lung cancer cells, were 1.9 and 2.37 µg/mL, respectively, which were two-fold lower than those of the fenugreek seed oil formulation. However, the IC50 value of the free drugs combination was not shown for reference.

## Nanostructured lipid carriers (NLCs)

NLCs are lipid-based nanoparticles comprising both solid and liquid lipids. This combination forms an amorphous lipid matrix accommodating high amounts of drugs and preventing drug leaching during storage and circulation [90]. Surfactants are used for NLC preparation to aid mixing of lipids and maintain colloidal stability of the particles [91].

Using the hot melting technique, Jeon et al. prepared RDV-loaded NLCs with glyceryl monostearate, Capmul MCM, and Myrj52, as solid lipid, liquid lipid, and surfactant, respectively [92]. The NLCs, intended for intravenous administration, sustained RDV release for over 48 h indicating its suitability for prolonged systemic circulation. RDV-loaded NLCs showed a tenfold higher in vitro antiviral activity than free RDV against SARS-CoV-2 in Vero E6 cells. This was attributed to a higher cell uptake and retention mediated by the NLCs as confirmed by measuring the intracellular level of RDV metabolite, GS-441524. A pharmacokinetic study in rats showed that NLCs increased RDV bioavailability by two folds. However, it is worth mentioning that although the authors report an almost 100% encapsulation efficiency, the RDV loading capacity corresponded to only 2% by weight. This adds to the cost of the drug and exposes patients to high excipient doses [78]. Similarly, another study employing glyceryl monostearate, stearic acid, and medium chain triglyceride for RDV loading in NLCs, following the hot emulsification and probe sonication technique, reported an approximately 80% encapsulation corresponding to 1.91% drug loading [93].

To maximize drug loading in NLCs, drug solubility in the used lipids is a critical parameter. For co-loading RDV and dexamethasone in NLCs, Capryol 90 showed a high RDV solubilizing power of 170 mg/g, however dexamethasone was poorly dissolved (9 mg/g) [94]. Thus, a cosurfactant, Transcutol P, was added to enhance dexamethasone solubility to 22 mg/g. Following ex vivo intratracheal administration, the NLCs sustained RDV retention in the lung tissue to 83% after 30 min, compared to 55% with RDV solution. This translated into a significant reduction in proinflammatory cytokine levels upon pulmonary delivery in an LPS inflammatory mouse model [94].

## Polymeric nanoparticles

### *Chitosan*

Chitosan, a biocompatible and biodegradable cationic polysaccharide, finds diverse applications in nano-based drug and gene delivery systems. Its cationic nature render it mucoadhesive, making it suitable for pulmonary applications by promoting adhesion and retention on the lung epithelial lining [95]. Chitosan is known for its antimicrobial and anticancer properties. Modifying the molecular weight, degree of acetylation, or chemically grafting chitosan yields derivatives with distinct charge distributions, physicochemical characteristics, and biological activities [96].

For RDV loading in chitosan-stabilized oil capsules, an internal oil core comprising Miglyol 812 N was encased in a lecithin layer complexed with chitosan via electrostatic interactions and hydrogen bonding between chitosan and lecithin [97]. To facilitate drug loading, RDV-SBECD complexes were utilized instead of free RDV, leveraging the negative charge of SBECD for robust electrostatic adsorption to the thick chitosan layer on the nanoparticles surface. An additional chitosan layer was deposited to enable the final adsorption of a negatively charged aptamer targeting the spike protein. By varying chitosan’s molecular weight, the highest RDV loading capacity of 5.6% was achieved with low molecular weight chitosan. However, medium molecular weight chitosan exhibited superior control over drug release and demonstrated potent inhibition of the HCoV-OC43 replication cycle, with an IC50 of 0.017 µg/mL compared with 12.5 µg/mL for RDV-SBECD [97].

### *Poly (lactic-co-glycolic acid) (PLGA)*

PLGA, an FDA-approved biocompatible and biodegradable polymer, stands as a highly effective material for drug delivery systems. It is frequently employed as a matrix for loading drugs in nanoparticles or as scaffolds for tissue engineering applications. PLGA demonstrates versatility in loading both hydrophilic and hydrophobic drugs, as well as macromolecules, with precise control over release kinetics [98, 99]. Functional groups can be conjugated to PLGA imparting additional functionalities to scaffolds or nanoparticles, such as receptor targeting or stimuli responsiveness [100, 101].

In a computational simulation study, RDV-loaded PLGA nanoparticles were designed with the aim for codelivery of RDV and lisinopril, an angiotensin-converting enzyme inhibitor associated with favorable lung outcomes in pneumonia patients [102, 103]. To co-load these distinct molecules with disparate solubilities, lisinopril was covalently grafted to PLGA, which was then employed to encapsulate RDV. Molecular docking elucidated the encapsulation of RDV within PLGA via intermolecular hydrogen bonding. Using dissipative particle dynamics simulations, the optimal carrier/drug ratios of 10:1 or 5:1 were determined, resulting in spherical nanoparticles with RDV in the hydrophobic core and lisinopril in the hydrophilic shell [102].

PLGA was also used for developing a long-acting injectable formulation for RDV [104]. Designed for subcutaneous self-administration, either manually or via an autoinjector, this formulation aimed to enhance convenience and reduce dosing frequency. RDV and PLGA were dissolved in N-methyl-2-pyrrolidone (NMP), a water-miscible biocompatible solvent that diffuses in aqueous environment upon injection, facilitating rapid phase separation of PLGA and soft gel depot formation. To optimize drug loading, the minimum volume of NMP capable of dissolving both RDV and PLGA without compatibility issues was used. PLGA formulations with different molecular weights and lactic/glycolic acid ratios were evaluated, with Resomer 752H at 15% demonstrating the highest drug loading capacity of 25%. This formulation exhibited sustained RDV release over 48 h, with a favorable burst release of 36%, ideal for an initial loading dose followed by a controlled release maintaining dosage regimen [8]. The injectability through 25G needles with low injection force was confirmed, indicating ease of administration. This formulation holds the promise of reducing dosing frequency from 5–8 intravenous doses to 2–3 subcutaneous injections, offering a more patient-friendly treatment approach.

### *Poloxamer and cyclodextrin*

Poloxamer 407 and β-cyclodextrin were used to encapsulate RDV in nanoparticles via a nanoprecipitation technique followed by ultrasonication [105]. β-cyclodextrin acted as a solubilizing agent, encapsulating RDV within its cavity forming an inclusion complex. Meanwhile, Poloxamer 407 served as a surfactant stabilizing the nanoparticles surface against aggregation. The resulting spherical particles had an average size of 500 nm and significantly enhanced dissolution of RDV in a pH 6.8 buffer simulating the nasal environment. In an OVA-albumin inflammatory mouse model, the RDV nanosuspension administered as a nasal spray significantly reduced the proinflammatory IL-4 mRNA plasma levels compared to intravenously administered RDV. Safety of the formulation was confirmed via histopathological analysis of the excised nasal tissue. However, RDV loading content in this formulation was limited to a maximum of 2.4% by weight, due to the substantial amount of cyclodextrin required for solubilization.

### *Dendritic polymers*

Dendritic polymers are hyperbranched polymers characterized by a 3D architecture that enables the formation of nanoparticles with a hollow cavity and outwardly branching chains. Their multivalency allows the encapsulation or conjugation of various drug molecules, as well as the incorporation of targeting moieties [106]. Commonly used dendrimers for drug delivery include PAMAM (polyamidoamine) and bis-MPA (2,2- bis(hydroxymethyl)propionic acid) [107]. Additionally, hybrid linear-dendritic block copolymers have been synthesized, which combine blocks of different chain topologies, merging the processability of linear polymers with the functionality of branched polymers [108].

In this context, RDV was encapsulated within a hyperbranched dendritic nano-scaffold made from a dendritic-linear-dendritic polymer consisting of two hyperbranched bis-MPA blocks (pseudo generation 4) linked via ester bonds to a central hydrophilic PEG chain (6 kDa). This was achieved using an O/W solvent evaporation method [109]. The polymer features 32 hydroxyl groups, which facilitates hydrogen bonding with RDV for efficient encapsulation along with van der Waals interaction. The PEG chains create a stealth coating around the nanoparticles, inducing steric repulsion despite a low surface charge. Notably, the RDV content reached 14%, which is comparatively high for other reported RDV nanocarriers. The combination of long PEG chains and an increased dendrimer generation contributes to a larger inner cavity of low density, accommodating high hydrophobic drug loadings [110].

## Inorganic nanoparticles

Inorganic nanoparticles such as zinc oxide, silver, and hydroxyapatite nanoparticles have found widespread use in biomaterials for promoting tissue regeneration and wound healing. Their application as carriers for systemic drug delivery has gained increasing attention in recent years. Compared to organic nanoparticles, inorganic ones are generally more stable and hydrophilic in nature, featuring tunable degradation rates [111]. Moreover, inorganic nanoparticles can possess exceptional physicochemical properties such as magnetic, thermal, and optical characteristics, that can impart functionality to the drug delivery system [112].

A comprehensive computational study explored the potential of zinc oxide nanoparticles as carrier for RDV, as they are biocompatible, inexpensive, and easy to prepare. By examining factors such as adsorption energies, charge transfer dynamics, and changes in electronic structure resulting from interactions between zinc oxide nanoparticles and RDV, stable and energetically favorable configurations conducive for drug delivery applications were identified [113].

Another inorganic nanomaterial considered for RDV delivery was mesoporous silica (mSiO_2_) [114]. The unique porous structure of mSiO_2_ enables high-capacity drug loading [115]. Fe(III) ions were incorporated into mSiO_2_ to enhance biodegradability and contrast efficacy for magnetic resonance imaging (MRI). Polydopamine coating was applied to the nanoparticles to prevent aggregation and ensure stability. Two loading strategies were explored: using RDV as a template during nanoparticle formation for simultaneous loading, or incubation with RDV post-nanoparticles formation for subsequent loading. The former method proved more effective, yielding encapsulation efficiencies of up to 70% corresponding to approximately 33% RDV content. The loading efficiency depended on the incubation duration and initial RDV concentration, as active sites in mSiO_2_ were gradually occupied with RDV until saturation was reached. The enhanced degradability due to Fe(III) doping was confirmed by comparing RDV release profiles between Fe(III)-doped mSiO2 and mSiO2, revealing 75% and 11% RDV release at physiological pH after 90 h, respectively [114].

Inorganic nanoparticles may demonstrate cytotoxic effects that are influenced by their size, shape, and surface chemistry. It is essential to evaluate how these characteristics impact cellular interactions and potential toxicity. Employing surface coatings can enhance the safety profile of these particles [116]. However, due to the potential for inhaled inorganic particles to provoke an inflammatory response in the lungs, their use as carriers for RDV in inhalable formulations is not advisable. This caution is particularly relevant given their prolonged retention time in the lungs and slow clearance, which could contribute to chronic pulmonary complications [117].

## Nebulizer inhalation

As SARS-CoV-2 causes pneumonia, the effective distribution of RDV in the lungs of COVID-19 patients is crucial to achieve potent antiviral efficacy while minimizing systemic toxicity. Nebulizer inhalation of reconstituted RDV solution has been proposed as a rapid and easy method for direct pulmonary delivery [8]. However, this approach is limited by the poor aqueous stability of RDV, characterized by a short half-life of < 1–2 h. Thus, nebulization duration should not exceed 30 min to avoid RDV hydrolysis into the polar NTP form that cannot effectively penetrate lung cells to manifest its antiviral effect. Furthermore, the potential lung tissue toxicity resulting from the high dose of SBECD present in the current injectable RDV formulation remains inadequately studied.

Ramsey et al. proposed an alternative nebulizer-compatible formulation for RDV, departing from the injectable form. By adopting a nanocrystal approach, they aimed to minimize the use of excipients [118]. Their study suggested delivering an RDV dose of 10 – 20 mg via nebulization, corresponding to 10 – 20% of the intravenous infusion dose. Utilizing the bottom-up nanoprecipitation technique, they developed RDV nanocrystals stabilized with Pluronic F-127, averaging 341 nm in size, followed by lyophilization with sucrose as a cryoprotectant. However, the nanocrystals precipitated after 30 min of aqueous reconstitution, with excessive foaming attributed to Pluronic F-127, leading to inconsistent drug output upon aerosolization. Therefore, they substituted Pluronic with a novel triblock copolymer with a Poly(2-oxazoline) backbone. The hydrophobic 2-*n*-butyl-2-oxazoline block was predicted to strongly adsorb onto the growing RDV crystals, while the hydrophilic 2-methyl-2-oxazoline blocks formed a protective brush stabilizing the resulting nanocrystals. This modification yielded larger crystals ranging from 400 to 500 nm that were stable for over 25 days in solution. The nanocrystal formulation exhibited a high RDV content of 95% and could be aerosolized via air jet nebulizer into respirable particles with a mass median aerodynamic diameter (MMAD) of around 5 µm, showing minimal foaming.

## Dry powder inhalation (DPI)

In addition to the considerable setup complexity and cost associated with nebulizer inhalation, the variability in lung delivery efficiency can impact the accurate dosing of RDV [119]. Moreover, the nebulizer holds a risk of contamination and could be a potential source for infection transmission [120]. An alternative strategy for RDV pulmonary delivery in an outpatient setting is to use DPI. DPI of RDV has been proposed as a mid-term strategy for treating COVID-19, especially if the disease transitions into a seasonal pattern. Compared to nebulizers, DPI supports higher stability, ease of self-administration, and a relatively lower cost [121]. They also require less coordination between actuation and inhalation, resulting in better pulmonary delivery efficiency [122].

DPI formulations typically consist of polymeric microparticles that can be non-porous, porous, or swellable, with sizes ranging from 1 to 5 µm. Ideally, these particles possess low density to facilitate deeper penetration through the respiratory tract [123]. Larger particles are sometimes used to evade phagocytosis and lung clearance, prolonging their therapeutic action. While nanoparticles have been explored for DPIs, their small size and density make them prone to being exhaled. They are also prone to aggregation upon aerosolization, negatively impacting their functionality especially in presenting surface targeting ligands. Thus, nanoparticles are often incorporated into larger carriers to aid in their aerosolization, lung deposition, and preservation of their properties until reaching target sites [124].

Thin film freezing (TFF) technique has been utilized to develop RDV as a DPI [125]. This emerging technique involves freezing the drug solution with a carrier system on a cryogenic surface forming a thin film, followed by sublimation of the solvent, resulting in highly porous, low density particles [126]. Sahakijpijarn et al. compared different solvent systems and found that 1,4-dioxane/water produced smaller nanoaggregates of RDV compared with acetonitrile/water. The higher viscosity of 1,4-dioxane/water restricts the molecular movement during rapid freeze drying, leading to a more uniformly dispersed system [127]. Various excipients were investigated as carriers for RDV to enhance its aerosolization, with Captisol® and leucine yielding smaller nanoaggregates compared to mannitol and lactose. These formulations maintained an amorphous structure of RDV, characteristic of the TFF process, ensuring a high dissolution rate of the resulting particles compared to unprocessed crystalline RDV [128]. The interaction between RDV and leucine, as observed through a depression in the melting point of leucine, could potentially benefit the stability of the formulation. Leucine-based formulations exhibited superior aerosol performance, with high fine particle fraction (FPF) and low MMAD.

A comparative pharmacokinetic study between leucine-based (RDV/Leu) and Captisol®-based (RDV/Cap) formulations in Syrian hamsters demonstrated that RDV/Cap achieved faster and greater absorption of RDV in the lungs, with higher concentrations in both lung tissue and plasma [129]. However, RDV/Leu showed promise by producing higher concentrations of the RDV metabolite, GS-441524, crucial for inhibiting virus replication in the lungs. Notably, both DPI formulations resulted in RDV plasma concentrations higher than previously reported in vitro EC_50_ values for RDV and GS-441524 [129].

The conventional spray drying technique has been also utilized to create DPI formulations of RDV. Spray drying offers a cost-effective method for producing free-flowing powders with uniform size and morphology [130]. In this process, leucine was used as a carrier for spray drying of RDV and disulfiram to achieve a combination DPI formulation [131]. The resulting powder had a spherical particle morphology with approximately 1% residual solvent, imperative for good powder flowability and effective aerosolization [132]. The spray-dried RDV, in the absence of leucine, exhibited an amorphous structure, while in the presence of leucine, it displayed a crystalline structure. Despite the lower dissolution rate of the crystalline form compared to the amorphous form, crystalline powders achieve higher lung deposition and are less likely to induce coughing [133, 134]. The DPI formulation demonstrated inhalable particle sizes, with an FPF and an emitted dose exceeding 60% and 88%, respectively. The DPI formulation showed minimal toxicity to lung cells in vitro and retained the antiviral activity against SARS-CoV-2 [131]. Similar results were obtained when RDV was co-spray dried with ebselen, an organoselenium compound of potent antimicrobial activity, using leucine as a carrier [135].

In an attempt to increase the dissolution rate of RDV upon inhalation, Wong et al. relied on the cocrystallization strategy prior to spray drying [136]. Salicylic acid (SA) was selected as a coformer, among other benzoic acid derivatives investigated, based on the formation of strong hydrogen bonding between the triazine ring in RDV and the carboxylic group in salicylic acid. SA possesses tenfold higher aqueous solubility than RDV thus their cocrystal would have an enhanced dissolution. The RDV cocrystal (RDV-SA) was prepared via liquid-assisted grinding and thermal annealing with a high RDV content (RDV/salicylic acid ratio of 10/2.3 by weight). Dissolution testing in simulated lung fluid showed approximately 70% RDV release from RDV-SA in 120 min compared to 5% release from unprocessed RDV. Salicylic acid was also reported to possess antiviral properties against SARS-CoV-2, thus a synergistic effect might be expected however was not tested in this study [137].

## Future perspectives and challenges

As the demand for effective treatments for COVID-19 and other emerging viral infections continues, reliance on innovative technologies for RDV delivery becomes important. Novel approaches such as enriching RDV within mesenchymal stem cell-derived exosomes and decorating these exosomes with anti-SARS antibodies present exciting avenues for enhanced RDV delivery [141]. This strategy aims to create a cell-free delivery system that specifically targets the microenvironment of coronavirus infection, thus promoting RDV’s antiviral and immunomodulatory effects. Additionally, the development of a novel cell-mimetic platform, known as a nanoviricide, designed to attack multiple coronavirus strains, has shown promise in encapsulating RDV. Composed of a PEG hydrophilic shell and a flexible micellar core of alkyl chain pendants, the nanoviricide effectively binds to free viral particles, encapsulating them and neutralizing their infectivity [142]. By loading RDV into this nanoviricide, RDV circulation half-life has increased, suggesting a potential delivery platform for augmented therapeutic potential [143].

Utilization of simulation methods, including molecular docking and molecular dynamics, is indispensable for advancing RDV formulation design. These techniques can facilitate the evaluation of complex RDV-carrier interactions under varying conditions [102, 144]. They can also enable in silico testing of ligand-target interactions, thus expediting the design process for effective drug delivery systems [145].

**Fig. 3:**
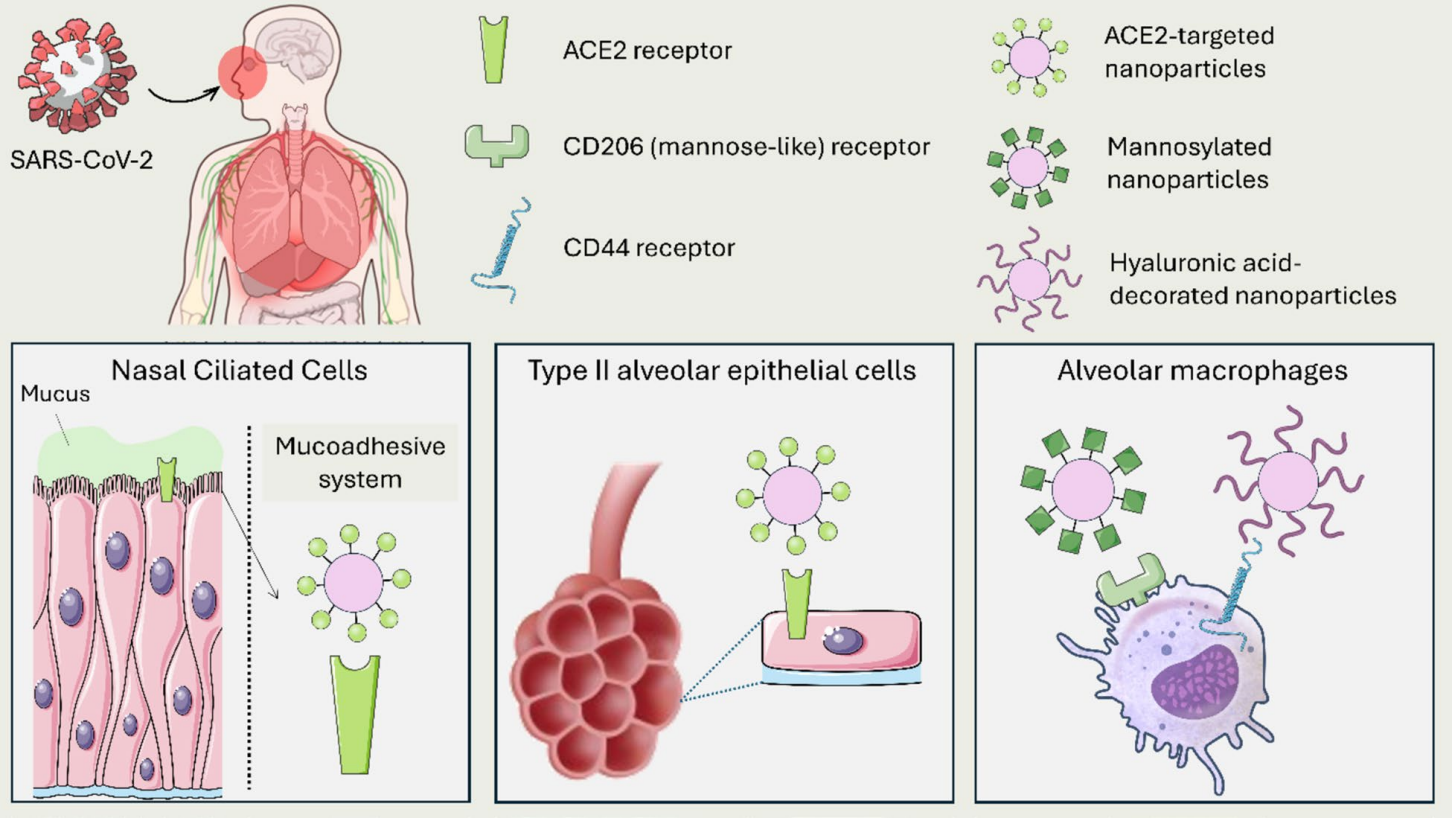
An illustration showing the key cell populations targeted by RDV along with potential targeting moieties for enhanced delivery

The targeting ability of nanoparticles can significantly enhance RDV delivery. Understanding the specific cell populations that RDV targets, illustrated in Fig. 3, can inform the choice of targeting moieties to enhance its delivery and efficacy. For instance, respiratory epithelial cells, as the primary entry point for SARS-CoV-2, are crucial for viral replication [146]. Therefore, engineered ligands or monoclonal antibodies targeting ACE2; highly expressed on surface of nasal ciliated cells and type II alveolar epithelial cells, could enhance RDV delivery to the infection target [147–149]. To successfully target the ciliated nasal epithelial cells, mucoadhesive nanoparticles should be used to overcome the ciliary clearance and increase the nasal residence time granting high cell uptake [150]. Furthermore, alveolar macrophages, which play a vital role in clearing respiratory pathogens, are another important target for RDV [151, 152]. Targeting the mannose-like receptor (CD206) on these immune cells could facilitate the effective delivery of RDV, with mannose-functionalized carriers enhancing macrophage uptake [153, 154]. CD44 is another surface receptor of alveolar macrophages that can be targeted with hyaluronic acid for enhanced nanoparticles uptake [155, 156].

Considering the demonstrated efficacy of discussed RDV combinations and the necessity for advanced delivery systems, introduction of RDV-based complex generics is anticipated. Complex generics could leverage the synergistic effects of multiple drugs with disparate physicochemical and pharmacokinetic properties, optimizing therapeutic outcomes. Advanced delivery systems are essential for accommodating these combinations, ensuring that each component is effectively delivered to the target site. However, for complex generics to successfully reach the market, sophisticated planning and optimization as well as deep understanding of regulatory and quality requirements are required [157].

Despite these promising advancements, several scalability challenges must be addressed. Although nanoparticles have been extensively studied at the laboratory scale, achieving precise control over formulation parameters for large-scale production remains complex. This difficulty can hinder the widespread availability of RDV during public health emergencies, such as the COVID-19 pandemic. Additionally, the cost effectiveness of the developed system is crucial for ensuring access of patients to critically needed medications in pandemic situations. However, advanced delivery systems utilizing advanced technologies and functional materials, such as targeting antibodies, may increase production cost substantially.

Regulatory hurdles also pose significant challenges. When changing the route of administration, for instance from intravenous RDV to inhalable RDV, or changing the dosage form, a New Drug Application (NDA) has to be submitted. This process can be lengthy and demands extensive preclinical and clinical data to demonstrate safety, efficacy, and pharmacokinetics. Compounding these challenges is the lack of uniform global regulations governing the use of nanoparticles, which can vary significantly between regulatory agencies/regions [158]. The diverse morphologies of nanoparticles also complicate the regulatory process, as each variation may require distinct safety assessments. Despite these challenges, navigating the regulatory landscape offers opportunities for researchers and pharmaceutical companies to secure intellectual property rights through innovative drug delivery systems that utilize proprietary technologies.

Moreover, toxicity concerns associated with advanced drug delivery systems must be carefully considered. For instance, nanoparticles may cross the BBB, increasing drug accumulation in the CNS. Given that RDV has shown neuromodulatory effects that could be associated with neurotoxicity, maintaining minimal CNS level of RDV is essential [159, 160]. However, the impact of viral persistence in the brain, which can lead to long COVID-related brain fog, underscores the necessity for targeted RDV delivery to the brain [82, 83]. Therefore, a comprehensive assessment of RDV concentrations in the brain is crucial to balance effective antiviral action against viral CNS persistence while mitigating potential neurotoxic effects. Similarly, the potential accumulation of nanoparticles in the reticuloendothelial system owing to size and charge considerations necessitate thorough investigation, especially considering RDV’s association with elevated liver enzymes levels and renal injury [161–163]. Additionally, improper disposal of nanoparticles can lead to environmental toxicity, raising further safety concerns.

Another important aspect to address is the development of in vivo models for effectively testing the efficacy of RDV delivery systems. Most of the reported literature lacks efficacy testing. Recent advancements have led to the creation of in vivo models that recapitulate the disease course and pathology of COVID-19 [164]. Additionally, for pharmacokinetic testing, it is important to measure the concentration of the pharmacologically active metabolite of RDV, NTP, as well as its parent nucleoside, GS-441524, rather than just RDV itself. Owing to RDV’s rapid distribution and conversion into NTP in peripheral blood mononuclear cells, its permeation into tissues might be limited, and therefore the correlation between plasma level and tissue level and thus efficacy might not be correct [8]. Focusing on metabolite concentration in lung tissue, the main site of infection, will provide a more accurate assessment of efficacy. Pharmacokinetic/Pharmacodynamic (PK/PD) modeling represents a powerful tool for predicting the efficacy, safety, toxicity, and optimal dosing schedules of RDV formulations, with a high throughput and reduced time and cost [145].

As we move forward, the integration of these insights into RDV formulation design and their application in clinical practice can lead to more effective management of COVID-19 and other viral infections, ultimately improving patient outcomes.

## Summary

The global surge in viral infections has heightened the demand for effective antiviral medications, making the development of drugs like RDV critical for immediate responses to emerging threats. RDV, recognized as the first FDA-approved treatment for COVID-19, is valued for its extensive antiviral activity compared to monoclonal antibodies. However, RDV therapy faces several challenges, including the necessity of intravenous infusion, which can be inconvenient, as well as toxicity risks associated with its formulation that may affect patients with renal or hepatic impairments. Additionally, RDV's limited accumulation in lung tissue hinders its effectiveness against respiratory infections. To address these issues, innovative drug delivery systems, such as nanoparticles and exosome-based platforms, are being explored to enhance RDV’s bioavailability, reduce toxicity, and improve targeting to affected tissues. Future research aims to optimize RDV delivery through novel technologies that can co-encapsulate RDV with repurposed molecules for synergistic activity and increase its deposition at the infection site using appropriate ligands. However, the path to market for complex generics combining RDV with other agents presents regulatory and scalability challenges, necessitating careful planning and compliance to ensure accessibility during public health emergencies. Furthermore, the review underscores the importance of developing effective in vivo models to accurately test RDV delivery systems, focusing on pharmacokinetics that assess the active metabolite (NTP) rather than RDV itself. Overall, while RDV shows significant promise in combating viral infections, overcoming these challenges will be crucial for maximizing its therapeutic potential and improving patient outcomes.

## Data Availability

No data is available.

## References

[1] Pardi N, Weissman D. Development of vaccines and antivirals for combating viral pandemics. Nat Biomed Eng. 2020;4(12):1128–33. 33293724 10.1038/s41551-020-00658-w PMC8336060

[2] Adamson CS, et al. Antiviral drug discovery: preparing for the next pandemic. Chem Soc Rev. 2021;50(6):3647–55. 33524090 10.1039/d0cs01118e

[3] Radoshitzky SR, et al. Expanded profiling of Remdesivir as a broad-spectrum antiviral and low potential for interaction with other medications in vitro. Sci Rep. 2023;13(1):3131. 36823196 10.1038/s41598-023-29517-9 PMC9950143

[4] Porter DP, et al. Remdesivir (GS-5734) Is Efficacious in Cynomolgus Macaques Infected With Marburg Virus. J Infect Dis. 2020;222(11):1894–901. 32479636 10.1093/infdis/jiaa290

[5] Lo MK, et al. Remdesivir (GS-5734) protects African green monkeys from Nipah virus challenge. Sci Trans Med. 2019;11(494):eaau9242. 10.1126/scitranslmed.aau9242 PMC6732787 31142680

[6] Mechineni A, et al. Remdesivir for the treatment of COVID 19: review of the pharmacological properties, safety and clinical effectiveness. Expert Opin Drug Saf. 2021;20(11):1299–307. 34338121 10.1080/14740338.2021.1962284

[7] Saha A, et al. Probable Molecular Mechanism of Remdesivir for the Treatment of COVID-19: Need to Know More. Arch Med Res. 2020;51(6):585–6. 32439198 10.1016/j.arcmed.2020.05.001 PMC7214327

[8] Sun D. Remdesivir for Treatment of COVID-19: Combination of Pulmonary and IV Administration May Offer Aditional Benefit. AAPS J. 2020;22(4):77. 32458279 10.1208/s12248-020-00459-8 PMC7250281

[9] Leegwater E, et al. Population Pharmacokinetics of Remdesivir and GS-441524 in Hospitalized COVID-19 Patients. Antimicrob Agents Chemother. 2022;66(6):e00254-e322. 35647646 10.1128/aac.00254-22 PMC9211420

[10] Sheahan TP, et al. Comparative therapeutic efficacy of remdesivir and combination lopinavir, ritonavir, and interferon beta against MERS-CoV. Nat Commun. 2020;11(1):019–13940. 10.1038/s41467-019-13940-6 PMC6954302 31924756

[11] Baldo BA. Immune- and Non-Immune-Mediated Adverse Effects of Monoclonal Antibody Therapy: A Survey of 110 Approved Antibodies. Antibodies. 2022;11(1):17. 10.3390/antib11010017. 35323191 10.3390/antib11010017 PMC8944650

[12] Sheahan TP, et al. Broad-spectrum antiviral GS-5734 inhibits both epidemic and zoonotic coronaviruses. Sci Transl Med. 2017;9(396):eaal3653. 10.1126/scitranslmed.aal3653. 10.1126/scitranslmed.aal3653 PMC5567817 28659436

[13] *FDA Approves First Treatment for COVID-19*. 22/10/2020; Available from: https://www.fda.gov/news-events/press-announcements/fda-approves-first-treatment-covid-19.

[14] Budi DS, et al. Remdesivir for pregnancy: A systematic review of antiviral therapy for COVID-19. Heliyon. 2022;8(1):08835. 10.1016/j.heliyon.2022.e08835 PMC8802673 35128114

[15] Solera JT, et al. Longitudinal outcomes of COVID-19 in solid organ transplant recipients from 2020 to 2023. Am J Transplant. 2024;24(7):1303–16. 38499087 10.1016/j.ajt.2024.03.011

[16] Chang H-Y, et al. Safety and effectiveness of remdesivir in hospitalized patients with COVID-19 and severe renal impairment: experience at a large medical center. Ann Med. 2024;56(1):2361843. 38830017 10.1080/07853890.2024.2361843 PMC11149583

[17] Khalil A, et al. Efficacy and Safety of Remdesivir in Hospitalized Pediatric COVID-19: A Retrospective Case-Controlled Study. Ther Clin Risk Manag. 2023;19:949–58. 38023628 10.2147/TCRM.S432565 PMC10680468

[18] Warren TK, et al. Therapeutic efficacy of the small molecule GS-5734 against Ebola virus in rhesus monkeys. Nature. 2016;531(7594):381–5. 26934220 10.1038/nature17180 PMC5551389

[19] Bodmer BS, et al. Remdesivir inhibits the polymerases of the novel filoviruses Lloviu and Bombali virus. Antiviral Res. 2021;192:105120. 34126139 10.1016/j.antiviral.2021.105120

[20] Konkolova E, et al. Remdesivir triphosphate can efficiently inhibit the RNA-dependent RNA polymerase from various flaviviruses. Antiviral Res. 2020;182:104899. 32763313 10.1016/j.antiviral.2020.104899 PMC7403104

[21] de Wit E, et al. Prophylactic and therapeutic remdesivir (GS-5734) treatment in the rhesus macaque model of MERS-CoV infection. Proc Natl Acad Sci U S A. 2020;117(12):6771–6. 32054787 10.1073/pnas.1922083117 PMC7104368

[22] Beigel JH, et al. Remdesivir for the Treatment of Covid-19 - Final Report. N Engl J Med. 2020;383(19):1813–26. 32445440 10.1056/NEJMoa2007764 PMC7262788

[23] Grein J, et al. Compassionate Use of Remdesivir for Patients with Severe Covid-19. N Engl J Med. 2020;382(24):2327–36. 32275812 10.1056/NEJMoa2007016 PMC7169476

[24] Dubert M, et al. Case report study of the first five COVID-19 patients treated with remdesivir in France. Int J Infect Dis. 2020;98:290–3. 10.1016/j.ijid.2020.06.093. 32619764 10.1016/j.ijid.2020.06.093 PMC7326458

[25] Igbinosa I, et al. Use of remdesivir for pregnant patients with severe novel coronavirus disease 2019. Am J Obstet Gynecol. 2020;223(5):768–70. 10.1016/j.ajog.2020.08.001. 32771381 10.1016/j.ajog.2020.08.001 PMC7410790

[26] Anderson J, et al. The use of convalescent plasma therapy and remdesivir in the successful management of a critically ill obstetric patient with novel coronavirus 2019 infection: A case report. Case Rep Womens Health. 2020;27:e00221. 10.1016/j.crwh.2020.e00221. 32426243 10.1016/j.crwh.2020.e00221 PMC7229947

[27] Goldman DL, et al. Compassionate Use of Remdesivir in Children With Severe COVID-19. Pediatrics. 2021;147(5):2020–047803. 10.1542/peds.2020-047803 33883243

[28] Rivera DR, et al. Utilization of COVID-19 Treatments and Clinical Outcomes among Patients with Cancer: A COVID-19 and Cancer Consortium (CCC19) Cohort Study. Cancer Discov. 2020;10(10):1514–27. 32699031 10.1158/2159-8290.CD-20-0941 PMC7541683

[29] Orf K, et al. Remdesivir during induction chemotherapy for newly diagnosed paediatric acute lymphoblastic leukaemia with concomitant SARS-CoV-2 infection. Br J Haematol. 2020;190(5):e274–6. 10.1111/bjh.17014. 32652563 10.1111/bjh.17014 PMC7404383

[30] Chen Y, et al. Remdesivir inhibits the progression of glioblastoma by enhancing endoplasmic reticulum stress. Biomed Pharmacother. 2023;157(114037):22. 10.1016/j.biopha.2022.114037 36427388

[31] Lutz JD, et al. Physiologically-Based Pharmacokinetic Modeling of Remdesivir and Its Metabolites to Support Dose Selection for the Treatment of Pediatric Patients With COVID-19. Clin Pharmacol Ther. 2021;109(4):1116–24. 33501997 10.1002/cpt.2176 PMC8014571

[32] Deb S, Reeves AA. Simulation of Remdesivir Pharmacokinetics and Its Drug Interactions. J Pharm Pharm Sci. 2021;24:277–91. 34107241 10.18433/jpps32011

[33] Eryilmaz IE, et al. The cytotoxic and antitumoral effects of Remdesivir, an antiviral RdRp inhibitor, on different cancer cells in vitro. Mol Cell Toxicol. 2023;20(3):649–60.

[34] Montazersaheb S, et al. COVID-19 infection: an overview on cytokine storm and related interventions. Virol J. 2022;19(1):92. 35619180 10.1186/s12985-022-01814-1 PMC9134144

[35] Gressens SB, et al. Remdesivir in combination with dexamethasone for patients hospitalized with COVID-19: A retrospective multicenter study. PLoS One. 2022;17(2):e0262564. 35176057 10.1371/journal.pone.0262564 PMC8853490

[36] Marrone A, et al. Remdesivir Plus Dexamethasone Versus Dexamethasone Alone for the Treatment of Coronavirus Disease 2019 (COVID-19) Patients Requiring Supplemental O2 Therapy: A Prospective Controlled Nonrandomized Study. Clin Infect Dis. 2022;75(1):e403–9. 35084022 10.1093/cid/ciac014 PMC8807307

[37] Ye ZW, et al. Beneficial effect of combinational methylprednisolone and remdesivir in hamster model of SARS-CoV-2 infection. Emerg Microbes Infect. 2021;10(1):291–304. 33538646 10.1080/22221751.2021.1885998 PMC7919885

[38] Kalil AC, et al. Baricitinib plus Remdesivir for Hospitalized Adults with Covid-19. N Engl J Med. 2021;384(9):795–807. 33306283 10.1056/NEJMoa2031994 PMC7745180

[39] Kojima Y, et al. Combination therapy with remdesivir and immunomodulators improves respiratory status in COVID-19: A retrospective study. J Med Virol. 2022;94(12):5702–12. 35916111 10.1002/jmv.28037 PMC9538820

[40] Hsu HY, et al. Remdesivir and cyclosporine synergistically inhibit the human coronaviruses OC43 and SARSCoV-2. Front Pharmacol. 2021;12:706901. 10.3389/fphar.2021.706901. 34483914 10.3389/fphar.2021.706901 PMC8409573

[41] Gidari A,et al. Synergistic activity of Remdesivir-Nirmatrelvir combination on a SARS-CoV-2 in vitro model and a case report. Viruses. 2023;15(7):1577. 10.3390/v15071577. 37515263 10.3390/v15071577 PMC10385213

[42] Chen T, et al. Synergistic Inhibition of SARS-CoV-2 Replication Using Disulfiram/Ebselen and Remdesivir. ACS Pharmacol Transl Sci. 2021;4(2):898–907. 33855277 10.1021/acsptsci.1c00022 PMC8009100

[43] Tan YL, et al. Combination treatment with remdesivir and ivermectin exerts highly synergistic and potent antiviral activity against murine coronavirus infection. Front Cell Infect Microbiol. 2021;11. 10.3389/fcimb.2021.700502. 10.3389/fcimb.2021.700502 PMC8362885 34395311

[44] Jeffreys LN, et al. Remdesivir-ivermectin combination displays synergistic interaction with improved in vitro activity against SARS-CoV-2. Int J Antimicrob Agents. 2022;59(3):31. 10.1016/j.ijantimicag.2022.106542 PMC8801767 35093538

[45] Schloer S, et al. Drug synergy of combinatory treatment with remdesivir and the repurposed drugs fluoxetine and itraconazole effectively impairs SARS-CoV-2 infection in vitro. Br J Pharmacol. 2021;178(11):2339–50. 33825201 10.1111/bph.15418 PMC8251190

[46] Kongsomros S, et al. Anti-SARS-CoV-2 activity of extracellular vesicle inhibitors: screening, validation, and combination with remdesivir. Biomedicines. 2021;9(9):1230. 10.3390/biomedicines9091230. 34572416 10.3390/biomedicines9091230 PMC8465755

[47] García-Crespo C, et al. Synergism between remdesivir and ribavirin leads to SARS-CoV-2 extinction in cell culture. Br J Pharmacol. 2024;181(15):2636–54. 38616133 10.1111/bph.16344

[48] Chiba S, et al. Co-administration of Favipiravir and the Remdesivir Metabolite GS-441524 Effectively Reduces SARS-CoV-2 Replication in the Lungs of the Syrian Hamster Model. mBio. 2021;13(1):03044–21. 10.1128/mbio.03044-21 PMC8805032 35100870

[49] Jang Y, et al. Comparison of antiviral activity of gemcitabine with 2’-Fluoro-2’-Deoxycytidine and combination therapy with remdesivir against SARS-CoV-2. Int J Mol Sci. 2021;22(4):1581. 10.3390/ijms22041581. 33557278 10.3390/ijms22041581 PMC7915419

[50] Tan YL, et al. Combination treatment with remdesivir and ivermectin exerts highly synergistic and potent antiviral activity against murine coronavirus infection. Front Cell Infect Microbiol. 2021;11. 10.3389/fcimb.2021.700502. 10.3389/fcimb.2021.700502 PMC8362885 34395311

[51] Gidari A, et al. Synergistic activity of Remdesivir-Nirmatrelvir combination on a SARS-CoV-2 in vitro model and a case report. Viruses. 2023;15(7):1577. 10.3390/v15071577. 10.3390/v15071577 PMC10385213 37515263

[52] Kongsomros S, et al. Anti-SARS-CoV-2 Activity of Extracellular Vesicle Inhibitors: Screening, Validation, and Combination with Remdesivir. Biomedicines. 2021;9(9):1230. 34572416 10.3390/biomedicines9091230 PMC8465755

[53] Ye Z-W, et al. Beneficial effect of combinational methylprednisolone and remdesivir in hamster model of SARS-CoV-2 infection. Emerg Microb Infect. 2021;10(1):291–304. 10.1080/22221751.2021.1885998 PMC7919885 33538646

[54] Nguyenla X, et al. Discovery of SARS-CoV-2 antiviral synergy between remdesivir and approved drugs in human lung cells. Sci Rep. 2022;12(1):18506. 36323770 10.1038/s41598-022-21034-5 PMC9628577

[55] Chiba S, et al. Co-administration of Favipiravir and the Remdesivir Metabolite GS-441524 Effectively Reduces SARS-CoV-2 Replication in the Lungs of the Syrian Hamster Model. mBio. 2022;13(1):e03044-21. 10.1128/mbio.03044-21 PMC8805032 35100870

[56] Jang Y, et al. Comparison of Antiviral Activity of Gemcitabine with 2′-Fluoro-2′-Deoxycytidine and Combination Therapy with Remdesivir against SARS-CoV-2. Int J Mol Sci. 2021;22(4):1581. 33557278 10.3390/ijms22041581 PMC7915419

[57] Xie J, Wang Z. Can remdesivir and its parent nucleoside GS-441524 be potential oral drugs? An in vitro and in vivo DMPK assessment. Acta Pharm Sin B. 2021;11(6):1607–16. 34221871 10.1016/j.apsb.2021.03.028 PMC8245906

[58] Aleissa Muneerah M et al., *New Perspectives on Antimicrobial Agents: Remdesivir Treatment for COVID-19.* Antimicrobial Agents and Chemotherapy, 2020;65(1). 10.1128/aac.01814-20 10.1128/AAC.01814-20 PMC7927874 33139290

[59] Schooley RT, et al. Rethinking Remdesivir: Synthesis, Antiviral Activity, and Pharmacokinetics of Oral Lipid Prodrugs. Antimicrob Agents Chemother. 2021;65(10):01155–221. 10.1128/AAC.01155-21 PMC8448143 34310217

[60] Lo MK, et al. Broad-Spectrum in vitro antiviral activity of ODBG-P-RVn: an orally-available, lipid-modified monophosphate prodrug of remdesivir parent nucleoside (GS-441524). Microbiol Spectr. 2021;9(3):e0153721. 10.1128/Spectrum.01537-21. 34817209 10.1128/Spectrum.01537-21 PMC8612139

[61] Luke DR, et al. Review of the basic and clinical pharmacology of sulfobutylether-beta-cyclodextrin (SBECD). J Pharm Sci. 2010;99(8):3291–301. 20213839 10.1002/jps.22109

[62] Santenna C et al., *The safety, tolerability and mortality reduction efficacy of remdesivir; based on randomized clinical trials, observational and case studies reported safety outcomes: an updated systematic review and meta-analysis.* Ther Adv Drug Saf, 2021;12(20420986211042517) 10.1177/20420986211042517 PMC8477695 34594487

[63] Humeniuk R, et al. Safety, Tolerability, and Pharmacokinetics of Remdesivir, An Antiviral for Treatment of COVID-19. Healthy Subjects Clin Transl Sci. 2020;13(5):896–906. 32589775 10.1111/cts.12840 PMC7361781

[64] Wee LE, et al. Detection of viable SARS-CoV-2 in deep respiratory specimens despite negative nasopharyngeal SARS-CoV-2 RT-PCR: Occult COVID-19 as an unsuspected cause of pulmonary infiltrates in immunocompromised patients. IDCases. 2022;30:e01611. 36032521 10.1016/j.idcr.2022.e01611 PMC9395297

[65] Chan HW, et al. Inhalable Nanoparticle-based Dry Powder Formulations for Respiratory Diseases: Challenges and Strategies for Translational Research. AAPS PharmSciTech. 2023;24(4):023–02559. 10.1208/s12249-023-02559-y PMC10072922 37016029

[66] Feng X, et al. Opportunities and Challenges for Inhalable Nanomedicine Formulations in Respiratory Diseases: A Review. Int J Nanomedicine. 2024;19:1509–38. 38384321 10.2147/IJN.S446919 PMC10880554

[67] Bakheit AH, et al. Remdesivir. Profiles Drug Subst Excip Relat Methodol. 2023;48:71–108. 37061276 10.1016/bs.podrm.2022.11.003 PMC9910426

[68] Chen R, et al. Antiviral Drug Delivery System for Enhanced Bioactivity, Better Metabolism and Pharmacokinetic Characteristics. Int J Nanomedicine. 2021;16:4959–84. 34326637 10.2147/IJN.S315705 PMC8315226

[69] Pagliarin LG, et al. In silico evidence of Remdesivir action in blood coagulation cascade modulation in COVID-19 treatment. World J Biol Chem. 2023;14(4):72–83. 37547340 10.4331/wjbc.v14.i4.72 PMC10401403

[70] Subhash C, et al. A Critical Assessment of Remdesivir. Current Drug Therapy. 2024;19(6):648–60.

[71] Cojocaru E, et al. *Nanoparticle-Based Drug Delivery Systems in Inhaled Therapy: Improving Respiratory Medicine*. Pharmaceuticals, 2024;17. 10.3390/ph17081059 10.3390/ph17081059 PMC11357421 39204164

[72] Li X, Peng X, Zoulikha M, Boafo GF, Magar KT, Ju Y, He W. Multifunctional nanoparticle-mediated combining therapy for human diseases. Signal Transduct Target Ther. 20249(1):1. 10.1038/s41392-023-01668-1. 10.1038/s41392-023-01668-1 PMC10758001 38161204

[73] Li Y, et al. Aromatized liposomes for sustained drug delivery. Nat Commun. 2023;14(1):6659. 37863880 10.1038/s41467-023-41946-8 PMC10589217

[74] Khan I et al., *9 - Liposome-based carrier systems and devices used for pulmonary drug delivery*, in *Biomaterials and Medical Tribology*, J.P. Davim, Editor 2013, Woodhead Publishing. 395–443

[75] Agudelo CW, et al. Alveolar lipids in pulmonary disease. A review Lipids Health Dis. 2020;19(1):020–01278. 10.1186/s12944-020-01278-8 PMC7268969 32493486

[76] Chono S, et al. Aerosolized liposomes with dipalmitoyl phosphatidylcholine enhance pulmonary insulin delivery. J Control Release. 2009;137(2):104–9. 19351549 10.1016/j.jconrel.2009.03.019

[77] Li J, et al. Liposomal remdesivir inhalation solution for targeted lung delivery as a novel therapeutic approach for COVID-19. Asian J Pharm Sci. 2021;16(6):772–83. 34703490 10.1016/j.ajps.2021.09.002 PMC8529908

[78] Taha MS, et al. Critical quality attributes in the development of therapeutic nanomedicines toward clinical translation. Drug Deliv Transl Res. 2020;10(3):766–90. 32170656 10.1007/s13346-020-00744-1

[79] Vartak R, et al. Aerosolized nanoliposomal carrier of remdesivir: an effective alternative for COVID-19 treatment in vitro. Nanomedicine. 2021;16(14):1187–202. 33982600 10.2217/nnm-2020-0475 PMC8117723

[80] Leekumjorn S, et al. The role of fatty acid unsaturation in minimizing biophysical changes on the structure and local effects of bilayer membranes. Biochim Biophys Acta. 2009;7(16):14. 10.1016/j.bbamem.2009.04.002 PMC2698950 19371719

[81] Fu Y, et al. EphA2-Receptor targeted PEGylated nanoliposomes for the treatment of BRAFV600E Mutated Parent- and Vemurafenib-Resistant Melanoma. Pharmaceutics 2019;11:504. 10.3390/pharmaceutics11100504 10.3390/pharmaceutics11100504 PMC6836218 31581483

[82] Mendes S, et al. Intranasal liposomal remdesivir induces SARS-CoV-2 clearance in K18-hACE2 mice and ensures survival. J Control Release. 2025;379:558–73. 39837387 10.1016/j.jconrel.2025.01.044

[83] eBioMedicine, *Post-COVID-19 condition, brain fog, and fatigue-what do we know now?* eBioMedicine, 2024;105 10.1016/j.ebiom.2024.105252 PMC11293575 38997174

[84] Wang Z, et al. Nose-to-Brain Delivery. J Pharmacol Exp Ther. 2019;370(3):593–601. 31126978 10.1124/jpet.119.258152

[85] Milkova V, et al. Remdesivir-Loaded Nanoliposomes Stabilized by Chitosan/Hyaluronic Acid Film with a Potential Application in the Treatment of Coronavirus Infection. Neurol Int. 2023;15(4):1320–38. 37987456 10.3390/neurolint15040083 PMC11340743

[86] El-Zahaby SA, et al. Zero-order release and bioavailability enhancement of poorly water soluble Vinpocetine from self-nanoemulsifying osmotic pump tablet. Pharm Dev Technol. 2018;23(9):900–10. 28540754 10.1080/10837450.2017.1335321

[87] Altamimi MA, et al. Development and optimization of self-nanoemulsifying drug delivery systems (SNEDDS) for curcumin transdermal delivery: an anti-inflammatory exposure. Drug Dev Ind Pharm. 2019;45(7):1073–8. 30987466 10.1080/03639045.2019.1593440

[88] Al Ashmawy AZG, Balata GF. Formulation and in vitro characterization of nanoemulsions containing remdesivir or licorice extract: A potential subcutaneous injection for coronavirus treatment. Colloids Surf B Biointerfaces. 2024;234(113703):9. 10.1016/j.colsurfb.2023.113703 38096607

[89] Kazi M, et al. Oral Bioactive Self-Nanoemulsifying Drug Delivery Systems of Remdesivir and Baricitinib: A Paradigmatic Case of Drug Repositioning for Cancer Management. Molecules. 2023;28(5):2237. 36903483 10.3390/molecules28052237 PMC10005540

[90] Khosa A, et al. Nanostructured lipid carriers for site-specific drug delivery. Biomed Pharmacother. 2018;103:598–613. 29677547 10.1016/j.biopha.2018.04.055

[91] Karn-Orachai K, et al. The effect of surfactant composition on the chemical and structural properties of nanostructured lipid carriers. J Microencapsul. 2014;31(6):609–18. 24861323 10.3109/02652048.2014.911374

[92] Jeon WJ, et al. Antiviral Lipid Nanocarrier Loaded with Remdesivir Effective Against SARS-CoV-2 in vitro Model. Int J Nanomedicine. 2023;18:1561–75. 37007987 10.2147/IJN.S391462 PMC10065008

[93] Amiri F, et al. An in vitro study for reducing the cytotoxicity and dose dumping risk of remdesivir via entrapment in nanostructured lipid carriers. Sci Rep. 2024;14(1):19360. 39169059 10.1038/s41598-024-70003-7 PMC11339451

[94] Chen C-W, et al. Pulmonary delivery of remdesivir and dexamethasone encapsulated nanostructured lipid carriers for enhanced inflammatory suppression in lung. J Drug Deliv Sci Technol. 2023;90:105144.

[95] Rasul RM, et al. A review on chitosan and its development as pulmonary particulate anti-infective and anti-cancer drug carriers. Carbohydr Polym. 2020;250(116800):18. 10.1016/j.carbpol.2020.116800 PMC7434482 33049807

[96] Alemu D, et al. Study on the Physicochemical Properties of Chitosan and their Applications in the Biomedical Sector. Int J Polym Sci. 2023;2023(1):5025341.

[97] Milkova V, et al. Chitosan-Based Nanocarriers for Delivery of Remdesivir. Sci Pharm. 2023;91(3):37.

[98] Español L, et al. Dual encapsulation of hydrophobic and hydrophilic drugs in PLGA nanoparticles by a single-step method: drug delivery and cytotoxicity assays. RSC Adv. 2016;6(112):111060–9.

[99] Ding D, Zhu Q. Recent advances of PLGA micro/nanoparticles for the delivery of biomacromolecular therapeutics. Mater Sci Eng, C. 2018;92:1041–60. 10.1016/j.msec.2017.12.036 30184728

[100] Zeeshan M, et al. Dual pH and microbial-sensitive galactosylated polymeric nanocargoes for multi-level targeting to combat ulcerative colitis. Asian J Pharm Sci. 2023;18(4):100831. 37588990 10.1016/j.ajps.2023.100831 PMC10425895

[101] Zhu J, et al. Mannose-Modified PLGA Nanoparticles for Sustained and Targeted Delivery in Hepatitis B Virus Immunoprophylaxis. AAPS PharmSciTech. 2019;21(1):13. 31807947 10.1208/s12249-019-1526-5

[102] Wu J, et al. Structure-aided ACEI-capped remdesivir-loaded novel PLGA nanoparticles: toward a computational simulation design for anti-SARS-CoV-2 therapy. Phys Chem Chem Phys. 2020;22(48):28434–9. 33305304 10.1039/d0cp04389c

[103] Mortensen EM, et al. Population-based study of statins, angiotensin II receptor blockers, and angiotensin-converting enzyme inhibitors on pneumonia-related outcomes. Clin Infect Dis. 2012;55(11):1466–73. 22918991 10.1093/cid/cis733 PMC3491858

[104] Patki M, et al. Self-injectable extended release formulation of Remdesivir (SelfExRem): A potential formulation alternative for COVID-19 treatment. Int J Pharm. 2021;597(120329):2. 10.1016/j.ijpharm.2021.120329 PMC7948064 33540028

[105] Mehmood Y, et al. Remdesivir nanosuspension for potential nasal drug delivery: determination of pro-inflammatory interleukin IL-4 mRNA expression and industrial scale-up strategy. J Nanopart Res. 2023;25(7):129. 10.1021/acsomega.3c02186 PMC10324090 37426214

[106] Wang J, et al. Dendrimer-based drug delivery systems: history, challenges, and latest developments. J Biol Eng. 2022;16(1):18. 35879774 10.1186/s13036-022-00298-5 PMC9317453

[107] Feliu N, et al. Stability and biocompatibility of a library of polyester dendrimers in comparison to polyamidoamine dendrimers. Biomaterials. 2012;33(7):1970–81. 22177621 10.1016/j.biomaterials.2011.11.054

[108] Abad M, et al. Supramolecular Functionalizable Linear-Dendritic Block Copolymers for the Preparation of Nanocarriers by Microfluidics. Polymers. 2021;13(5):684. 10.3390/polym13050684. 33668750 10.3390/polym13050684 PMC7956801

[109] Halevas E, et al. Remdesivir-loaded bis-MPA hyperbranched dendritic nanocarriers for pulmonary delivery. J Drug Deliv Sci Technol. 2022;75(103625):10. 10.1016/j.jddst.2022.103625 PMC9364662 35966803

[110] Kojima C, et al. Synthesis of polyamidoamine dendrimers having poly(ethylene glycol) grafts and their ability to encapsulate anticancer drugs. Bioconjug Chem. 2000;11(6):910–7. 11087341 10.1021/bc0000583

[111] Unnikrishnan G, et al. Exploration of inorganic nanoparticles for revolutionary drug delivery applications: a critical review. Discover Nano. 2023;18(1):157. 38112849 10.1186/s11671-023-03943-0 PMC10730791

[112] Liong M, et al. Multifunctional Inorganic Nanoparticles for Imaging, Targeting, and Drug Delivery. ACS Nano. 2008;2(5):889–96. 19206485 10.1021/nn800072t PMC2751731

[113] Kurban M, Muz İ. Investigating the potential use of (ZnO)60 nanoparticle as drug delivery system for chloroquine, hydroxychloroquine, favipiravir, and remdesivir. Mater Today Commun. 2024;38:108488.

[114] Arkaban H, et al. Fabrication of Fe(III)-doped mesoporous silica nanoparticles as biocompatible and biodegradable theranostic system for Remdesivir delivery and MRI contrast agent. Inorg Chem Commun. 2023;150:110398. 36644526 10.1016/j.inoche.2023.110398 PMC9827735

[115] Bharti C, et al. Mesoporous silica nanoparticles in target drug delivery system: A review. Int J Pharm Investig. 2015;5(3):124–33. 26258053 10.4103/2230-973X.160844 PMC4522861

[116] Wang X, et al. Use of Coated Silver Nanoparticles to Understand the Relationship of Particle Dissolution and Bioavailability to Cell and Lung Toxicological Potential. Small. 2014;10(2):385–98. 24039004 10.1002/smll.201301597 PMC4001734

[117] Uskoković V. Lessons from the history of inorganic nanoparticles for inhalable diagnostics and therapeutics. Adv Coll Interface Sci. 2023;315:102903. 10.1016/j.cis.2023.102903 37084546

[118] Ramsey JD, et al. Nanoformulated Remdesivir with Extremely Low Content of Poly(2-oxazoline)-Based Stabilizer for Aerosol Treatment of COVID-19. Macromol Biosci. 2022;22(8):26. 10.1002/mabi.202200056 PMC9347370 35526106

[119] Longest W, et al. Devices for improved delivery of nebulized pharmaceutical aerosols to the lungs. J Aerosol Med Pulm Drug Deliv. 2019;32(5):317–39. 10.1089/jamp.2018.1508. 31287369 10.1089/jamp.2018.1508 PMC6781258

[120] Reychler G, et al. Nebulization: A potential source of SARS-CoV-2 transmission. Res Med Res. 2020;78:100778. 10.1016/j.resmer.2020.100778 PMC7399661 32763845

[121] Gardenhire DS, et al. *A Guide to Aerosol Delivery Devices.* 2013

[122] Ibrahim M, et al. Inhalation drug delivery devices: technology update. Med Dev. 2015;8:131–9. 10.2147/MDER.S48888 PMC4334339 25709510

[123] Shahin HI, Chablani L. A comprehensive overview of dry powder inhalers for pulmonary drug delivery: challenges, advances, optimization techniques, and applications. J Drug Deliv Sci Technol. 2023;84:104553.

[124] Sung JC, et al. Nanoparticles for drug delivery to the lungs. Trends Biotechnol. 2007;25(12):563–70. 10.1016/j.tibtech.2007.09.005. 17997181 10.1016/j.tibtech.2007.09.005

[125] Sahakijpijarn S et al., *Development of Remdesivir as a Dry Powder for Inhalation by Thin Film Freezing.* Pharmaceutics, 2020;12(11) 10.3390/pharmaceutics12111002 PMC7690377 33105618

[126] Pardeshi SR et al., *Progress on Thin Film Freezing Technology for Dry Powder Inhalation Formulations.* Pharmaceutics, 2022;14(12) 10.3390/pharmaceutics14122632 PMC9784462 36559129

[127] Moon C, et al. Processing design space is critical for voriconazole nanoaggregates for dry powder inhalation produced by thin film freezing. J Drug Deliv Sci Technol. 2019;54:101295.

[128] Overhoff KA, et al. Novel ultra-rapid freezing particle engineering process for enhancement of dissolution rates of poorly water-soluble drugs. Eur J Pharm Biopharm. 2007;65(1):57–67. 16987642 10.1016/j.ejpb.2006.07.012

[129] Sahakijpijarn S et al., (2021) *In vivo pharmacokinetic study of remdesivir dry powder for inhalation in hamsters.* Int J Pharm X, 2021;3(100073) 10.1016/j.ijpx.2021.100073 PMC8683664 34977555

[130] ElKasabgy NA, et al. Respiratory Tract: Structure and Attractions for Drug Delivery Using Dry Powder Inhalers. AAPS PharmSciTech. 2020;21(7):238. 32827062 10.1208/s12249-020-01757-2

[131] Saha T, et al. Inhalable dry powder containing remdesivir and disulfiram: Preparation and in vitro characterization. Int J Pharm. 2023;645(123411):12. 10.1016/j.ijpharm.2023.123411 37703955

[132] Zhu K, et al. Analysis of the influence of relative humidity on the moisture sorption of particles and the aerosolization process in a dry powder inhaler. J Aerosol Sci. 2008;39(6):510–24.

[133] Carvalho SR, et al. Characterization and pharmacokinetic analysis of crystalline versus amorphous rapamycin dry powder via pulmonary administration in rats. Eur J Pharm Biopharm. 2014;88(1):136–47. 10.1016/j.ejpb.2014.05.008. 24859653 10.1016/j.ejpb.2014.05.008

[134] Sahakijpijarn S, et al. Post-inhalation cough with therapeutic aerosols: Formulation considerations. Adv Drug Deliv Rev. 2020;166:127–41. 10.1016/j.addr.2020.05.003 32417367

[135] Saha T et al., *Spray-Dried Inhalable Microparticles Combining Remdesivir and Ebselen against SARS-CoV-2 Infection.* Pharmaceutics, 2023;15(9) 10.3390/pharmaceutics15092229 PMC10535576 37765198

[136] Wong SN, et al. Synthesis of the first remdesivir cocrystal: design, characterization, and therapeutic potential for pulmonary delivery. Int J Pharm. 2023;640:122983. 37121494 10.1016/j.ijpharm.2023.122983

[137] Geiger N, et al. Acetylsalicylic Acid and Salicylic Acid Inhibit SARS-CoV-2 Replication in Precision-Cut Lung Slices. Vaccines. 2022;10(10):1619. 36298484 10.3390/vaccines10101619 PMC9609675

[138] Chan HW, et al. Integrated continuous manufacturing of inhalable remdesivir nanoagglomerate dry powders: Design, optimization and therapeutic potential for respiratory viral infections. Int J Pharm. 2023;644:123303. 37579825 10.1016/j.ijpharm.2023.123303

[139] Fouad SA, et al. Novel inhalable nano-based/microparticles for enhanced sustained pulmonary delivery of remdesivir - A patient malleable treatment for coronaviruses infection: In vitro aerosolization, cytotoxicity assays and antiviral activity studies. J Drug Deliv Sci Technol. 2024;101:106196.

[140] Szente L, et al. Sulfobutylether-beta-cyclodextrin-enabled antiviral remdesivir: Characterization of electrospun- and lyophilized formulations. Carbohyd Polym. 2021;264:118011. 10.1016/j.carbpol.2021.118011 PMC8025548 33910715

[141] Daneshi N, et al. 47D11 antibody-engineered exosomes for targeted delivery of remdesivir in patients with COVID-19: dream or principle? (A Critical Editorial Study). Eurasian J Med. 2022;54(3):310–2. 10.5152/eurasianjmed.2022.21116. 35950831 10.5152/eurasianjmed.2022.21116 PMC9797761

[142] Chakraborty A, et al. Mechanism of Antiviral Activities of Nanoviricide’s Platform Technology based Biopolymer (NV-CoV-2). AIMS Public Health. 2022;9(2):415–22. 35634020 10.3934/publichealth.2022028 PMC9114783

[143] Chakraborty A et al., *Nanoviricide’s platform technology based NV-CoV-2 polymer increases the half-life of Remdesivir in vivo.* bioRxiv, 2021: 2021.11.17.468980

[144] Zhang Y, et al. Molecular docking assisted exploration on solubilization of poorly soluble drug remdesivir in sulfobutyl ether-tycyclodextrin. AAPS Open. 2022;8(1):9. 10.1186/s41120-022-00054-5. 35498163 10.1186/s41120-022-00054-5 PMC9035334

[145] Salahshoori I, et al. Simulation-based approaches for drug delivery systems: Navigating advancements, opportunities, and challenges. J Mol Liq. 2024;395: 123888.

[146] Ravindra NG, et al. Single-cell longitudinal analysis of SARS-CoV-2 infection in human airway epithelium identifies target cells, alterations in gene expression, and cell state changes. PLoS Biol. 2021;19(3):e3001143. 10.1371/journal.pbio.3001143. 33730024 10.1371/journal.pbio.3001143 PMC8007021

[147] Jia H, et al. Targeting ACE2 for COVID-19 Therapy: Opportunities and Challenges. Am J Respir Cell Mol Biol. 2021;64(4):416–25. 33296619 10.1165/rcmb.2020-0322PS PMC8008810

[148] Ahn JH et al., *Nasal ciliated cells are primary targets for SARS-CoV-2 replication in the early stage of COVID-19.* J Clin Invest, 2021;131(13) 10.1172/JCI148517 PMC8245175 34003804

[149] Carcaterra M, Caruso C. Alveolar epithelial cell type II as main target of SARS-CoV-2 virus and COVID-19 development via NF-Kb pathway deregulation: A physio-pathological theory. Med Hypotheses. 2021;146:110412. 33308936 10.1016/j.mehy.2020.110412 PMC7681037

[150] Taha MS, et al. Topical Administration of Verapamil in Poly(ethylene glycol)-Modified Liposomes for Enhanced Sinonasal Tissue Residence in Chronic Rhinosinusitis: Ex Vivo and In Vivo Evaluations. Mol Pharm. 2023;20(3):1729–36. 36744718 10.1021/acs.molpharmaceut.2c00943 PMC10629233

[151] Huot N, et al. SARS-CoV-2 viral persistence in lung alveolar macrophages is controlled by IFN-γ and NK cells. Nat Immunol. 2023;24(12):2068–79. 37919524 10.1038/s41590-023-01661-4 PMC10681903

[152] Wang Z, Li S, Huang B. Alveolar macrophages: Achilles’ heel of SARS-CoV-2 infection. Signal Transduct Target Ther. 2022;7(1):242. 35853858 10.1038/s41392-022-01106-8 PMC9295089

[153] Zlotnikov ID, Kudryashova EV. Mannose receptors of alveolar macrophages as a target for the addressed delivery of medicines to the lungs. Russ J Bioorg Chem. 2022;48(1):46–75. 10.1134/S1068162022010150.

[154] Bera H et al. *Mannose-Decorated Solid-Lipid Nanoparticles for Alveolar Macrophage Targeted Delivery of Rifampicin*. Pharmaceutics, 2024;16, 10.3390/pharmaceutics16030429 10.3390/pharmaceutics16030429 PMC10975733 38543323

[155] Dong Y, et al. CD44 loss disrupts lung lipid surfactant homeostasis and exacerbates oxidized lipid-induced lung inflammation. Front Immunol. 2020;11:29. 10.3389/fimmu.2020.00029. 10.3389/fimmu.2020.00029 PMC7002364 32082314

[156] Mattheolabakis G, et al. Hyaluronic acid targeting of CD44 for cancer therapy: from receptor biology to nanomedicine. J Drug Target. 2015;23(7–8):605–18. 26453158 10.3109/1061186X.2015.1052072

[157] Patil S et al., *Current Regulatory Framework and Challenges for the Approval of Complex Generics in the US and the EU.* Current Indian Science, 2024;2(1)

[158] Ali F, Neha K, Parveen S. Current regulatory landscape of nanomaterials and nanomedicines: A global perspective. J Drug Deliv Sci Technol. 2023;80:104118.

[159] Gulati G, Kelly BD. Does remdesivir have any neuropsychiatric adverse effects? Irish J Psychol Med. 2021;38(4):313–4. 10.1017/ipm.2020.67 32641172

[160] Krisanova N, et al. Amphiphilic anti-SARS-CoV-2 drug remdesivir incorporates into the lipid bilayer and nerve terminal membranes influencing excitatory and inhibitory neurotransmission. Biochim Biophys Acta Biomembr. 2022;1864(8). 10.1016/j.bbamem.2022.183945. 10.1016/j.bbamem.2022.183945 PMC9023372 35461828

[161] El-Haroun H, et al. Impact of Remdesivir on the kidney and Potential Protective Capacity of Granulocyte-Colony Stimulating Factor Versus Bone Marrow Mesenchymal Stem Cells in Adult Male Albino Rats. Egypt J Histol. 2022;45(2):338–58.

[162] Lin K et al., *Acute Liver Failure Secondary to Remdesivir in the Treatment of COVID-19.* ACG Case Reports Journal, 2022;9(10) 10.14309/crj.0000000000000866 PMC9534366 36212242

[163] van Laar SA, et al. Liver and kidney function in patients with Covid-19 treated with remdesivir. Br J Clin Pharmacol. 2021;87(11):4450–4. 33763917 10.1111/bcp.14831 PMC8251044

[164] Rosa RB, et al. In Vitro and In Vivo Models for Studying SARS-CoV-2, the Etiological Agent Responsible for COVID-19 Pandemic. Viruses. 2021;13(3):379. 33673614 10.3390/v13030379 PMC7997194

